# Decoding regulatory landscape of somatic embryogenesis reveals differential regulatory networks between *japonica* and *indica* rice subspecies

**DOI:** 10.1038/srep23050

**Published:** 2016-03-14

**Authors:** Yuvraj Indoliya, Poonam Tiwari, Abhisekh Singh Chauhan, Ridhi Goel, Manju Shri, Sumit Kumar Bag, Debasis Chakrabarty

**Affiliations:** 1 Council of Scientific and Industrial Research - National Botanical Research Institute (CSIR-NBRI), Rana Pratap Marg, Lucknow-226001, India; 2 Academy of Scientific and Innovative Research (AcSIR), Anusandhan Bhawan, 2 Rafi Marg, New Delhi-110 001, India

## Abstract

Somatic embryogenesis is a unique process in plants and has considerable interest for biotechnological application. Compare to *japonica*, *indica* rice has been less responsive to *in vitro* culture. We used Illumina Hiseq 2000 sequencing platform for comparative transcriptome analysis between two rice subspecies at six different developmental stages combined with a tag-based digital gene expression profiling. Global gene expression among different samples showed greater complexity in *japonica* rice compared to *indica* which may be due to polyphyletic origin of two rice subspecies. Expression pattern in initial stage indicate major differences in proembryogenic callus induction phase that may serve as key regulator to observe differences between both subspecies. Our data suggests that phytohormone signaling pathways consist of elaborate networks with frequent crosstalk, thereby allowing plants to regulate somatic embryogenesis pathway. However, this crosstalk varies between the two rice subspecies. Down regulation of positive regulators of meristem development (i.e. *KNOX*, *OsARF5*) and up regulation of its counterparts (*OsRRs*, *MYB*, *GA20ox1*/*GA3ox2*) in *japonica* may be responsible for its better regeneration and differentiation of somatic embryos. Comprehensive gene expression information in the present experiment may also facilitate to understand the monocot specific meristem regulation for dedifferentiation of somatic cell to embryogenic cells.

Rice (*Oryza sativa* L.) acts as a model crop to study plant development and functional genomic studies due to its comparatively small genome size (430 Mb)^1^, better-syntenic closeness with genome of other cereals and relatively efficient *Agrobacterium*-mediated transformation. Integration of biotechnology through genetic engineering, an alternative for conventional breeding methods requires an efficient *in vitro* culture protocols for rice improvement^2^. Among the cereals, rice and maize are largely responsive for tissue culture and are capable of regeneration in *in vitro* conditions^3^.

Somatic embryogenesis is the process of developing bipolar structures that derived from haploid or diploid somatic cells and formed through an embryological stage without fusion of gametes that are not connected to the primary vascular tissues of the mother calli. It is a unique process in plants and has considerable interest for biotechnological application such as clonal propagation, production of synthetic seeds and genetic transformation^4^,^5^. Somatic embryogenesis in integration with classical breeding programs and molecular biology techniques provides a valuable tool to enhance the genetic improvement of crop species^4^. Likewise it is also useful in studying embryo development processes and several plant physiological aspects^6^,^7^,^8^. In rice, it is the most common regeneration pathway and has been mainly obtained from mature seeds^9^,^10^.

*Oryza sativa* contains two major subspecies: the sticky, short grained *japonica* variety, and the non-sticky, long-grained *indica* variety. The *indica* sub-species of rice have been less responsive to *in-vitro* culture as compared to *japonica*^11^,^12^. Moreover, considerable variations in regeneration efficiency among a multiplicity of rice cultivars, especially between these sub-species also exist based on research with model varieties such as Nipponbare (*japonica*), PB-1 and IR64 [*indica*]^13^,^14^. As a model species with completely sequenced genomes of two subspecies, *indica* and *japonica*, rice holds a unique position to study questions regarding genomic network regulating developmental events of somatic embryogenesis and regeneration. The polyphyletic hypothesis postulates that the *indica* subspecies and the *japonica* subspecies each originated from different common wild rice ancestral populations^15^,^16^ which may be an important factor of their regeneration ability during *in vitro* culture. Majority of plant development related genomic approaches mainly focused on *in vivo* embryogenesis (Zygotic embryogenesis) and its respective developmental events^17^,^18^,^19^,^20^. However, very few reports about *in vitro* developmental studies in plants are available^21^,^22^,^23^. Totipotency, the competence of whole plant regeneration, depends on the genetic potential of a particular plant and therefore, it is very difficult to understand the molecular mechanisms of plant regeneration. With the aim of understanding and clarifying the mechanism of differential regeneration processes of two rice subspecies, we embarked on studies to identify genes playing important roles in the signal transduction pathway involved in the somatic embryogenesis and regeneration process. Our approach is based on the use of comparative trascriptome profiling of Nipponbare (*japonica*) and PB-1 (*indica*) at six different developmental stages of somatic embryogenesis and regeneration (Stage 1–6) using the Illumina Hiseq 2000 transcriptome sequencing platform.

## Result and Discussion

### Establishment of an efficient somatic embryogenesis and regeneration protocol in *japonica* and *indica* subspecies

**Figure 1:**
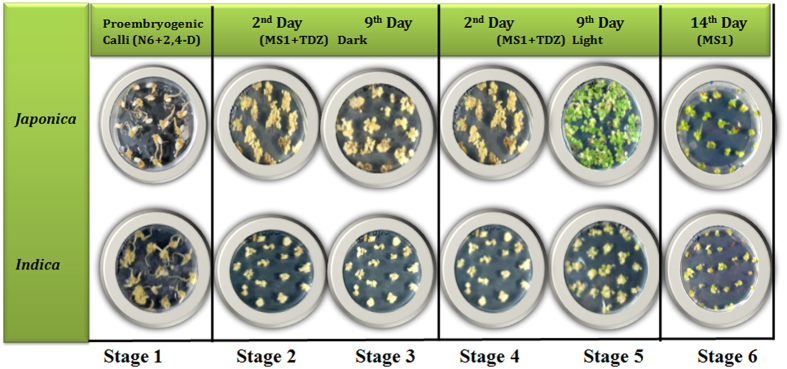
Comparative somatic embryogenesis and regeneration efficiency among *japonica* and *indica* rice subspecies showing very high somatic embryogenesis and regeneration potential of *japonica* compare to *indica*.

Mature seeds of *japonica* and *indica* rice sub-species showed proembryogenic callus induction on N6 medium supplemented with 3 mg l^−1^ 2,4-D. Most of the seed explants exhibited callus initiation after 7–10 days from the scutellar region. There is clear distinction between the callus morphology between the two subspecies. The calli of *japonica* subspecies exhibited whitish friable calli while *indica* subspecies developed nodular creamish-white proembryogenic calli after 30 days of culture (Fig. S1). The 2,4-D induced embryogenic calli were examined at different time points for shoot initiation frequency in MS+TDZ and MS-1 (without TDZ) containing regeneration medium as described in materials and methods. As shown in Fig. S1, high frequency of greening was observed in MS+TDZ media as compared to MS-1. TDZ induced high frequency regeneration in both *japonica* and *indica* is in agreement with previous reports in several plants including rice^10^,^24^,^25^. Further, in order to analyze differential somatic embryogenesis and regeneration pattern between *japonica* and *indica*, calli of both subspecies were again kept under observation in MS+TDZ media. We observed significant phenotypic difference in terms of green spots, an indicator of regeneration, between *japonica* and *indica* sub-species (Fig. 1, Table S1). Phenotypes were consistently kept under observation upto 15 days. Almost all calli (≥90%) of *japonica* sub-species turned green upto 9^th^ day PLT (Post Light Treatment) whereas comparatively very less greening was observed in *indica* sub-species (Table S1). Moreover, *japonica* sub-species show early greening compare to *indica* (Fig. 1, Table S1). The green sectors turned into shoot meristems and further differentiation takes place upon removing TDZ from the medium. The results are in agreement of previous reports about low somatic embryogenesis and regeneration efficiency of *indica* sub-species where they have shown four representative varieties of *indica* subspecies, i.e. PB1, IR64, CSR10 and Swarna to be less responsive for somatic embryogenesis^13^,^14^. Based on such phenotypic observation, six sequential developmental checkpoints during somatic embryogenesis and regeneration were selected (Fig. 1) for further analysis.

### Generation and analysis of the RNA-seq data set shows differential transcript abundance

To systematically investigate the dynamics and differential analysis of transcriptome over *in vitro* development, RNA-seq libraries of *japonica* and *indica* subspecies at six different developmental stages, as described in previous section, were prepared following steps mentioned in Fig. S2. The fastq summary is provided in Table 1. On average 88% of total data passed >= 30 Phred score. The low quality bases were trimmed from the reads. The read passed the quality filtering is further used for reference based alignment using Tophat. To further investigate sequencing efficiency, percentage read coverage in different time points of *japonica* and *indica* subspecies based on reference mapping were also analyzed. As shown in Fig. S3, maximum percentage of read coverage lying between 90–100 indicates accuracy of the sequencing data for subsequent analysis. Further, uniquely mapped reads were used to estimate normalized gene expression level as Fragments Per Kilo base of transcript per Million mapped reads (FPKM). To reduce the influence of transcription noise, genes from the *japonica* and *indica* filtered gene set (FGS) were included for analysis only if their FPKM values were ≥1.

Schatz *et al.*^26^ discussed about novel genes among three different strains of rice; *Oryza sativa*, *aus* and *indica.* To explore novel genes among both subspecies, stage specific unique genes of *japonica* and *indica* subspecies were analyzed as described in materials and methods. In total, we identified 3416 and 789 novel genes expressed in at least 1 of the 6 samples in *japonica* and *indica* subspecies respectively. The stage specific distribution of these genes is revealed by a Venn diagram (Fig. S4) which shows that 963 and 72 novel genes were common among all six developmental stages of *japonica* and *indica* subspecies respectively. The number of novel genes identified in *japonica* during the different developmental stages was higher as compared to *indica*. FPKM based expression of *japonica* and *indica* specific genes in different developmental stages are given in Table S2A,B Result indicates that compare to *japonica*, *indica* subspecies has lesser number of novel genes. Data also support previous report about strain specific novel genes in rice^26^.

**Figure 2:**
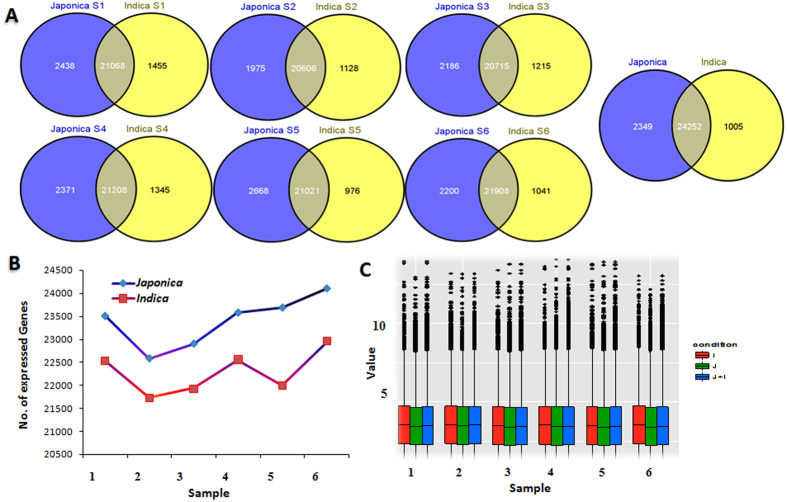
Analysis of global gene expression among different samples showing overrepresentation of genes in *japonica* subspecies compare to *indica*. (**A**) Venn diagram of the differential expressed genes detected among *japonica* and *indica* showing common and unique genes identified among both subspecies in sequential developmental stages. (**B**) Total Number of genes expressed in each of the samples. (**C**) Comparison of FPKM based expression levels of genes detected among *japonica* and *indica* subspecies. I*, indica* expressed *japonica* not expressed (i.e. FPKM values of all six stages of *indica*); J, j*aponica* expressed *indica* not expressed (i.e. FPKM values of all six stages of *japonica*); J+I, Both *japonica* and *indica* expressed (i.e. FPKM values of all six stages of both *indica* and *japonica* collectively).

In total, we identified 27,606 genes expressed in at least 1 of the 12 samples. The distribution of these genes is revealed by a Venn diagram (Fig. 2A), which shows that 24,252 genes were common among *japonica* and *indica* subspecies. The number of genes identified in *japonica* during the sequential developmental stages was higher as compared to *indica* and these were found to be overrepresented in both subspecies during the later developmental stages. Although the expression pattern was dynamic as it was higher during initial stage followed by reduced expression in a couple of subsequent stages and finally higher expression in later developmental stages (Fig. 2B). Further, to analyze overall expression pattern of whole transcriptome data,the FPKM based median expression pattern was studied by generating Box plot using ggplot2 vs 1.0.1 (https://cran.r-project.org/web/packages/ggplot2/index.html). As shown in Fig. 2C, median expression level found to be almost similar among both subspecies indicating overall similarity in expression pattern between both subspecies. The overrepresentation of genes in *japonica* may be associated with greater molecular complexity due to its different wild rice ancestral populations, i.e. *Oryza rufipogon* and *Oryza nivara* for *japonica* and *indica*, respectively^22^,^23^ and that might be responsible for differential pattern of somatic embryogenesis and regeneration between both subspecies.

### Global gene expression pattern shows diversity among different development stages

To gain insight into the relationships among the different samples based on expression pattern, principal component analysis (PCA) was performed, which graphically displayed the transcriptional signatures and developmental similarity. The first component (84.64% variance explained) separated samples based on subspecies identity (Fig. S5A). The second component (6.14% variance explained) discriminated different developmental stages of *japonica* and *indica* subspecies respectively (Fig. S5A). As shown in Fig. S5A, clear variation was observed in stage 1 of *japonica* and *indica* subspecies indicating major transcriptional difference in initial developmental stage (i.e. proembryogenic calli). Stage 2 of *japonica* showing similarity with same stage in *indica*. Similarly, Stage 3 of both subspecies also behaves alike. Further, Stage 4 and 5 behaves in a different way, as both consecutive stages behave closely within *japonica* and *indica* separately. Final stage (stage 6) of both subspecies showed similar expression pattern collectively. Dynamic expression pattern of clusters in both *japonica* and *indica* separately grouped well along the axis of developmental time (Fig. S6A,B) according to phenotypic observation described initially. Both samples of stage 6 were present in one node of primary cluster, while rest of the samples was grouped in another node of the same cluster, which correspond to different phases of development and differentiation of somatic embryos among both subspecies (Fig. S6A,B). This is consistent with the previous reports about callus induction, somatic embryo development and its further regeneration events and then switching to apical meristem formation and differentiation events^10^. Expression differences in initial stage between both subspecies indicate major differences in proembryogenic callus induction phase of *in vitro* embryogenesis in rice (Fig. S5B). Further analysis may give better assessment of such possibility. Almost similar expression pattern in later stages of somatic embryogenesis and regeneration between *japonica* and *indica* also strengthen the possibility of major differences in initial stage i.e. proembryogenic callus induction phase. Stage 4 and 5 of both subspecies are in accordance with the starting phase of chloroplast biogenesis and regeneration in rice upon light treatment^10^. Such observation can be correlated with our establish protocol, described previously in initial results of phenotypic difference, showing visible difference in Stage 4 and 5 between *japonica* and *indica* subspecies (Fig. 1). Since major differences were found in proembryogenic callus induction phase (Stage 1) as indicated by PCA and hierarchal clustering, this phase may serve as key regulator to observe differences between both subspecies. Results also confirm that the expression data following characteristic somatic embryo development phases and therefore may further explored as valuable insights about respective changes in the transcriptomic datasets of different stages.

### Transcripts of functional significance are dynamically and differentially expressed during different developmental stages

**Figure 3:**
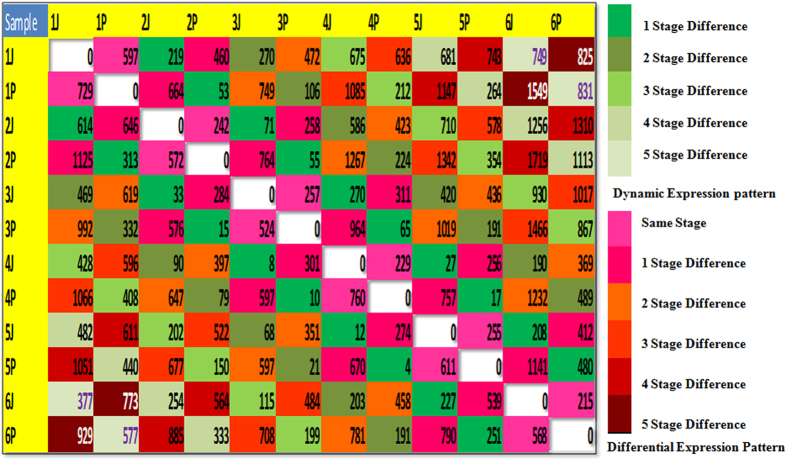
Dynamic and Differential expression profile of genes preferentially expressed in each sample as compared with others in a sample-wise comparison showing greater complexiciy in number of genes as stage difference increases. Genes having two or greater fold change as compared with the other sample are given. For the genes in each cell, there is preferential expression in the column sample than in the row sample. Stage difference indicates possible sequential gaps among respective stages.

**Figure 4:**
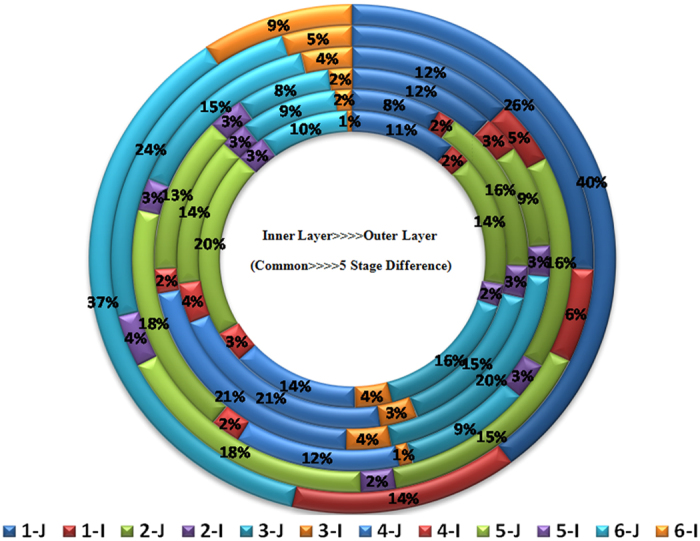
Differential expression pattern with number of genes showing unique expression with different expression abundances in various stages between *japonica* and *indica* subspecies of rice. The stage specific unique genes are represented by three or greater fold change in the sample of interest compare to other. Different colors showing respective stages while inner to outer layer indicate stage difference in ascending order.

**Figure 5:**
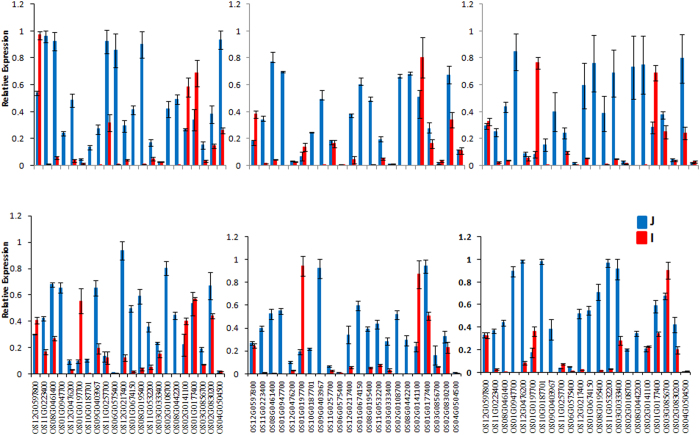
Quantitative Real Time PCR of selected genes in different developmental stages of somatic embryogenesis in rice. Relative expression for all genes, in all samples were calculated using delta-delta CT method. Bars show the mean of triplicate samples and error bars represent the SD. Pooled triplicate samples of harvested RNA as described in materials and methods were used for Real Time validation.

For differential gene expression analysis, transcript abundance is a well–accepted method as it is an indicator of gene abundance and has been applied in several studies^27^,^28^. The results of differential gene expression analysis using NGS technologies have been found to be accurate and highly correlated with other methods such as real–time PCR analysis and microarray analysis^29^. For transcripts abundance analysis of rice, the high-quality reads from individual samples were mapped to the transcripts and the number of reads mapped was normalized by the FPKM method. The FPKM method corrects for biases in total gene exon size and normalizes for the total read sequences obtained in each sample library. We classified the gene expression in five categories (very low, low, moderate, high, and very high), arbitrarily based on the FPKM value of each transcript in different samples (Fig. S7). The largest fraction of transcripts showed moderate expression (FPKM 10–50) followed by low expression (FPKM 3–10) and very low expression (FPKM 1–3) in all samples. Small fractions (5–10%) of transcripts were expressed at high levels (FPKM 50–100) and very high levels (FPKM > 100) in different samples (Fig. S7). Furthermore, we identified transcripts expressed preferentially in each sample analyzed via sample wise comparison (Fig. 3). For this, we normalized the number of reads uniquely mapped to each transcript by FPKM method as described previously. Figure 3 shows the number of genes that have significant preferential expression (3-fold or greater) between two stages. We found very high variability in fraction of genes that are preferentially expressed among the two samples analyzed in this study. The largest number of dynamic expression genes showed preferential expression in J6 as compared with J2, followed by I6 compare to I2 whereas least expression was observed in I4 compare to I5 followed by 3J compare with 4J. Similarly largest number of differential expression genes showed preferential expression in J6 as compared with I2, followed by J6 compare to I1 whereas least expression was observed in I6 compare to J6 followed by 4I compare with 4J. Overall Fig. 3 indicate that as stage difference between different samples increases, number of differentially expressing genes (DEGs) also increases gradually in almost all cases. This pattern was true for both *japonica* and i*ndica* subspecies. Possible explanation of such an observation may be due to diverse molecular signaling according to respective developmental events and morphological changes during somatic embryogenesis and regeneration in rice. TDZ mediated somatic embryogenesis and regeneration in rice comprises of four crucial developmental and morphological shifts as shown earlier in Fig. 1. These shifts are 2,4-D mediated seed to proembryogenic calli induction; proembryogenic calli to TDZ mediated somatic embryo development in dark; TDZ mediated light induced regeneration followed by further differentiation in basal media (MS-1). Majority of differentially expressed genes in Stage 6 compare to Stage 2 in both subspecies may be due to difference in these crucial developmental shifts as in Stage 6, shoot formation and further differentiation of aerial organs occur which is a completely different phenomenon from Stage 2 as it is the starting phase of TDZ mediated embryogenesis from proembryogenic calli. Least differential trascripts modulation in middle stages (Stage 3 and 5) between two developmental shifts indicating almost similar expression pattern in a given condition. One of the major developmental and morphological shifts during somatic embryogenesis is light induced chlorophyll biogenesis and Shoot Apical Meristem (SAM) differentiation. Majority of differentially expressing genes throughout above mentioned shifts indicating a complex molecular network regulating somatic embryo development in both the subspecies. The identification of preferentially expressed genes within dynamics of both *japonica* and *indica* separately will help to gain insight into the gene functions and thereby the biological processes, whereas differential expression pattern between both sub-species may give us a clue to identify gene network involve in distinct somatic embryogenesis and regeneration among both subspecies of rice. Furthermore, in order to check novel expression pattern of genes during different developmental stages, we identified unique genes expressed specifically in a single sample with at least three fold changes in the sample of interest and zero in other (Fig. 4, Fig. S7, Table S3A–C). Dynamic expression of both sub-species individually showed that as the intra stage difference increases, fraction of genes exhibited specific expression also increases gradually (Fig. S8, Table S3A,B). Largest fractions of genes expressed specifically with variable abundance were found in Stage 6 followed by Stage 1 and Stage 4 whereas smallest portions were observed in stage 3 followed by Stage 2 and Stage 5 in both *japonica* and *indica* subspecies (Fig. S8, Table S3A,B). Interestingly, Stage 1 and 6 of *indica* subspecies behaves differently as compared to similar stages in *japonica* as unique genes was higher in Stage 1 of *indica* whereas in case of *japonica* same was true for Stage 6. This pattern was in accordance with the PCA and hierarchical clustering result where Stage 1 of *japonica* and *indica* shows dissimilarity with each other. Differential expression between both subspecies was also analyzed which showed overrepresentation of unique genes in *japonica* over *indica* subspecies among all the developmental stages (Fig. 4, Table S3C). Over representation of uniquely expressing genes in *japonica* may be associated with greater molecular complexity in *japonica* with response to somatic embryogenesis and regeneration as compared to *indica.* These genes may play specific role in the biology of various developmental stages during somatic embryogenesis and further developmental events in rice. The results have been validated by quantitative real-time PCR analysis of some of the selected genes (Fig. 5). A very good correlation was analyzed by real-time PCR analysis with respect to transcriptomic data (Fig. 5, Table S4). These results further confirm the potential of NGS technologies to quantify gene expression.

### Gene annotation and functional categorization indicate diversity among different developmental stages

Functional categorization of significant differential expressing genes from stage wise enrichment analysis was performed through Singular Enrichment Analysis (SEA) using agriGO tool [http://bioinfo.cau.edu.cn/agriGO/] for *japonica* and *indica* subspecies (Fig. S9, Table S5). Singular Enrichment Analysis between both subspecies showed significant differential expression of hormone responsive stimuli (auxin, cytokinin, and ethylene), reproduction, transcription factors, extracellular region related genes in Stage 1 whereas ion transmembrane transporter related genes were differentially regulated specifically in Stage 2 (Fig. S9). Further, receptor mediated activity were differentially regulated in Stage 1 and Stage 6. One of the receptor protein kinase, *CLAVATA1* reported to play an important role in SAM maintenance during *WUS*-*CLAVATA* mediated signaling in its maintenance and differentiation^30^. In addition to it, photosynthesis and its related genes were found to be differentially expressed in Stage 4 and 5. Dynamic expression pattern of photosynthesis related genes in Stage 4 and 5 only is an agreement of previous report of TDZ mediated chlorophyll biogenesis (appearance of green sectors) in presence of light^10^,^24^,^25^. Expression pattern of genes related to development, transcriptional regulation, regulation of biological process, and signaling were also significantly different between both subspecies. Role of auxin and cytokinin for induction and expression of embryogenesis has already been reported^24^. Ethylene also reported to play role in somatic embryogenesis induction^31^. Several transcription factors also play vital role in somatic embryogenesis in many plants^32^,^33^. Stage wise coordinated differential expression pattern of the genes, directly or indirectly involve in *in vitro* embryogenesis and regeneration may assist in further understanding of molecular network regulating somatic embryogenesis among both subspecies in our future study.

**Figure 6:**
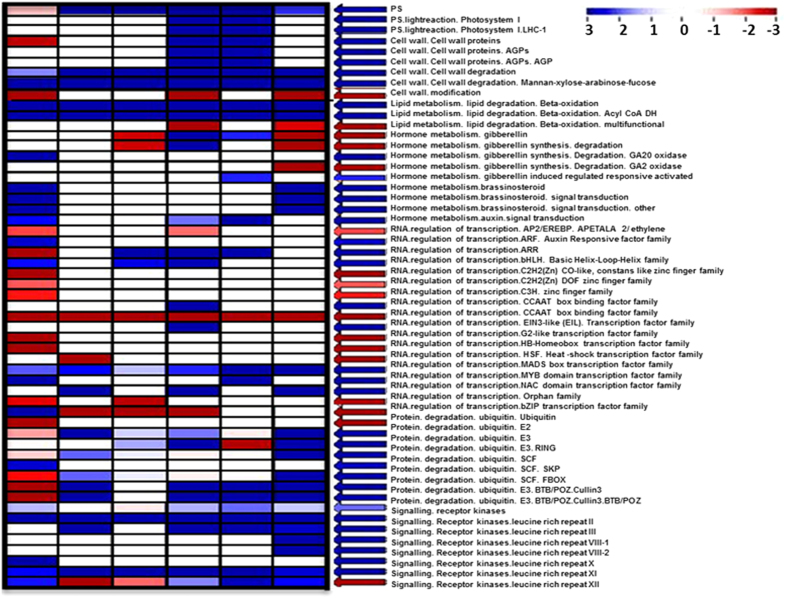
Enrichment analysis of specific differentially expressing genes (DEGs) using PageMan tool showing stage specific up and down regulation of representing gene in *japonica* subspecies of rice compare to *indica*.

Further, PageMan analysis tool was used to obtain a statistics-based overview of enriched functional categories among different developmental stages of both subspecies. Stage specific DEGs showed enriched pathways related to photosynthesis, Cell wall degradation and lipid degradation (beta-oxidation) activity in *japonica* sub-species (Fig. 6). In our study, as evident from morphological difference, most of the photosynthesis related pathways was found to be up regulated in Stage 4 of *japonica* sub-species, which plays a role in somatic embryo maturation in plants^34^,^35^. Role of cell wall degradation in somatic embryogenesis has also been reported^36^. In hormone metabolism, brassinosteroid (BR) signal transduction mediated pathway were significantly over-represented in *japonica* subspecies at Stage 1 and Stage 6 (Fig. 6). BR cross signaling with numerous other hormones in regulating many developmental processes in plants has already been reported^37^. We next analyzed the differentially expressing TFs between both sub-species. As shown in Fig. 6, *MYB, AP2/EREBP, NAC, bHLH* and *Orphan* families were found to be up regulated in *japonica* sub-species. Differential expression pattern of several transcription factors including MYB, AP2, NAC and bHLH have already been reported to involve in meristem development in Maize^38^. In our data, five *MYB* family transcription factor related genes (*OS06G0348800*, *OS04G0665600*, *OS03G0325500*, *OS07G0685300*, and *OS09G0299200*) were found to be up regulated in almost all developmental stages of *japonica* compare to *indica*. Interestingly one *MYB* related gene, *OS12G0572201*, an orthologous of maize *Rough sheath2* and Arabidopsis *ASYMMETRIC LEAVES1 (AS1)* showing higher expression in *japonica* compared to *indica* subspecies. The product of this gene encodes a *myb* type protein that represses *KNOX* expression. In Arabidopsis, Maize and Snapdragon, this gene is termed as *ARP* proteins [*ASYMMETRIC LEAVES1 (AS1)*, *Rough sheath2*, *PHANTASTICA*, respectively]^39^,^40^,^41^. *ARP* and *KNOX* gene expression distinguishes leaf founder cell from meristem cell fate in the shoot apex. Additionally, three members of the *AP2/ERF* transcription factor, *BABY BOOM* [*OS01G0899800* and *OS04G0504500*] and *PLETHORA 7* (*OS03G0770700*) were overrepresented in proembryogenic callus stage (Stage 1) of *japonica* compare with the same in *indica*. *BABY BOOM (BBM)* was initially identified in *Brassica napus* where it expressed preferentially during embryogenesis and development of seeds^32^. It is reported to induce embryonic callus in dicots like *Brassica* and *Nicotiana*^32^,^33^. *Glycine max BBM* (*GmBBM*) mediated embryonic callus induction in *Arabidopsis* seedlings has also been reported^42^, suggesting that the function of *BBM* in promoting embryogenesis or embryonic callus formation. Over expression of *BBMs* in initial developmental stage of *japonica* may be associated with higher proembryonic callus induction thereby greater somatic embryogenesis and regeneration compare to its counterpart. Overexpression of *PLETHORA 7* in *japonica* especially after TDZ treatment gives indication about its probable role in somatic embryogenesis and regeneration in rice. Expression pattern of *NAC* domain containing transcription factors was also analyzed (Table S7). Among all *NAC* family members; almost four genes were showing up regulation in *japonica* in all stages. Involvement of *NAC* transcription factors in development of the SAM has already been reported^43^. Although *NAC* family proteins are involved in various processes only a few have been characterized. Members of this family reported to involve in delimiting organs during embryonic, floral, and vegetative development^43^. Further characterization is needed to gain insights into involvement of *NAC* family members in somatic embryogenesis and pattern formation. We also noticed that most of the auxin signaling mediated transcription factor families like *Aux/IAA* and *ARF* were differentially expressed among *japonica* and *indica* sub-species (Table S6). Interestingly, one of *cullin* repeat like domain containing gene *Os05g0369900* found to be up regulated in proembryogenic callus stage of *japonica* compare to *indica* (Table S7). *Cullin3*, an *E3 ubiquitin*, responsible for auxin mediated *Aux/IAA* degradation thereby induction of auxin responsive elements in callus^44^. Overexpression of *cullin* may be correlated with greater auxin signaling and its downstream regulation of somatic embryogenesis in *japonica* compare to *indica*. Additionally, genes related to *leucine rich repeat receptor kinases* were overrepresented in *japonica* sub-species. Expression pattern of genes of *leucine rich repeats* family was analyzed and we found four genes [*OS10G0358200, OS12G0500500, OS11G0673900, OS11G0605100*] (Table S7) showing overexpression uniquely in *japonica* calli compared to *indica*. *Leucine-rich repeat (LRR)* domain fused to a central nucleotide binding domain (*NB-LRR* proteins) and collectively called the *NB-ARC* domain^45^. Role of *NB-ARC* domain containing receptor kinases related to somatic embryogenesis reported in several plant species^46^,^47^,^48^. The overrepresentation of such genes and respective pathways in *japonica* sub-species discussed above may be responsible for better somatic embryogenesis in *japonica* sub-species as compared to *indica.*

### Cluster analysis of transcript abundances in 6 different developmental stages of *japonica* and *indica* subspecies

**Figure 7:**
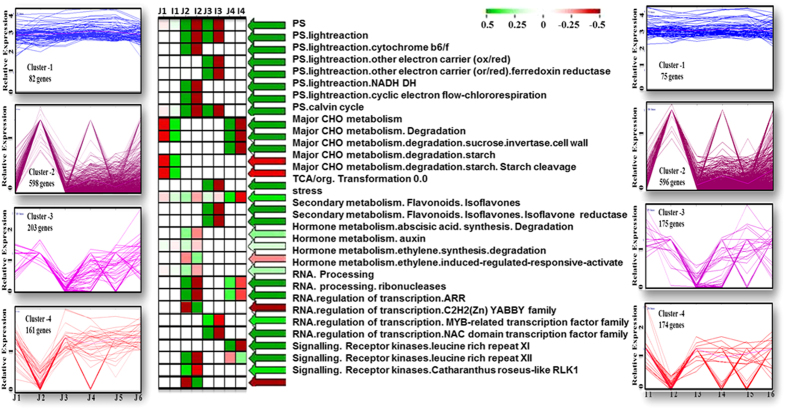
Cluster analyses of transcript abundances for 4 successive time points of each subspecies in (a) *japonica* and (b) *indica* subspecies. (c) Functional category enrichment among the four somatic embryo development and differentiation related clusters through MapMan bins. Red: significantly over-represented; green: significantly underrepresented.

PageMan analysis of eight selected clusters (Fig. 7 and Figs S10 and S11) showed that auxin induced regulation was more prominent at initial stages in the *japonica* subspecies compared to *indica*. Similar pattern was true for ethylene induced regulation. Auxin induced somatic embryogenesis via regulation of upstream and downstream mediators of *ARF*-*Aux/IAA* signaling pathway in several plants including rice has already been reported^8^,^24^. Expression pattern of auxin binding proteins (*ABPs*) have been reported to up regulate in callus inducing tissues of seed legumes^8^. Over representation of auxin induced regulation in *japonica* may be correlated with higher degree of somatic embryogenesis of *japonica* sub-species compare to *indica*. Further molecular characterization of differentially expressing genes may confirm the exact regulation between both sub-species. Role of ethylene towards somatic embryogenesis have been investigated in many ways. Precursor of ethylene, ethylene 1-aminocyclopropane-1-carboxylic acid (*ACC*), has been reported to improve somatic embryo induction and further development of globular embryos in *Leucojum aestivum*^31^. Cluster analysis also revealed up regulation of transcription factors including *YABBY* and *MYB* whereas cytokinin mediated *ARR* transcriptional regulation found to be under represented in *japonica* subspecies compared to *indica* (Fig. 7). The products of *YABBY, specially YAB1 and YAB3* genes of rice have been reported to play key role in meristem development through gibberellic acid mediated signaling^49^. One of the *YABBY* gene, *YAB1 (OS07G0160100*) found to be up regulated in *japonica* after TDZ and light mediated induction [Stage 4 and 5] (Table S6). Expression of *YAB1* increases gradually as it was low in initial stages of both subspecies followed by higher expression pattern in later developmental stages (Table S5). In fact, dynamic expression pattern of other members of *YABBY* family followed almost similar pattern (Table S6). Rice *YAB1* is induced by GA (Gibberellic Acid) and it acts as feedback regulator of GA biosynthesis in the shoot through repressing the GA biosynthetic gene *GA3ox2*^50^. In our data *GA3ox2* (*OS01G0177400*) was up regulated in *japonica* as compared to *indica*. Another GA biosynthetic gene *GA20ox1* (*OS03G0856700*) also found to be up regulated in *japonica* compare to *indica*. *GA20ox1* is inhibited by *KNOX1*, a positive regulator of SAM maintenance^51^. Cytokinin mediated *KNOX* proteins regulate the balance between GAs and CKs (Cytokinins) in shoot apical meristem. In the SAM, *KNOX* proteins decreases GA levels by negatively regulating its biosynthetic gene *GA 20-oxidase1* (*GA20ox1*), thereby decreasing GA level inside SAM to maintain cells in undifferentiated state^51^,^52^. Thus, KNOX genes are considered to inhibit cell differentiation in the SAM by decreasing and increasing the amount of GA and CK, respectively. Cytokinins stimulate cell division through activating GA catabolic gene expression, whereas GAs promotes cell elongation to maintain proper balance between differentiation and replenishment of cells in the SAM^52^. Increase level of GA biosynthetic genes in *japonica* may trigger cell differentiation and regeneration in a better way as compared to *indica*.

### Hormonal regulation during six different time points in *japonica* and *indica* sub-species

**Figure 8:**
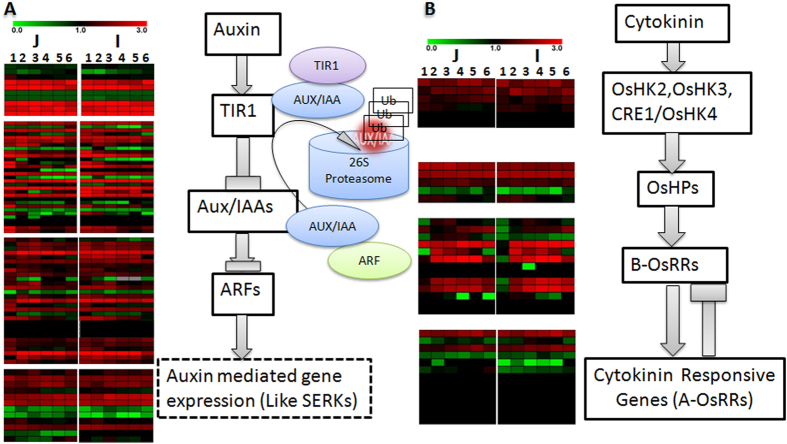
Putative pathway for hormone signaling in rice. All the enzymes found in this study related to different steps are shown between the reactions catalyzed. Expression of different transcripts related to these enzymes in *japonica* and *indica* is shown by heatmap. (**A**) Auxin signaling, (**B**) Cytokinin signaling Red: significantly under-represented; blue: significantly over represented.

As mentioned earlier about role of various phytohormones in somatic embryogenesis and regeneration in rice. To gain insights into various aspects of hormonal regulation (i.e. biosynthesis, catabolism, signaling), FPKM based transcripts expression pattern was analyzed. As shown in Fig. 8 (Table S8), auxin mediated signaling shows several genes were differentially expressed between *japonica* and *indica* subspecies. This pattern was followed by cytokinin mediated signaling pathway. Auxin signaling regulated via *ARF*-*Aux/IAA* mediated loop where *Aux/IAA* works as repressor of *ARFs* (Auxin responsive Factors)^53^, whereas cytokinin followed two component response regulators mediated pathway for signaling and response^54^,^55^. In our data, several auxin responsive genes showed differential expression pattern among both subspecies. one of the auxin responsive gene *ARF5* (*OS02G0141100*) also known as ‘*MONOPTEROS (MP)’* was found to be down regulated in all developmental stages in *japonica* subspecies compared to *indica* (Table S8). Whereas most of the cytokinin mediated type A response regulators (*OsRRs* Type A) were over-represented in initial developmental stage of *japonica*. TDZ mediated up regulation of type A response regulators have already been reported^10^. Interestingly, among all *OsRRs*, *OsRR3* (*OS02G0830200*) shows overexpression in *japonica* among all developmental stages compare to *indica* (Table S8). Expression pattern of *OsHPs* (Histidine phosphotransferases) and *OsHKs* (*Histidine Kinases*), initial mediators of cytokinin signaling were almost similar among both subspecies indicating similar response of initial cytokinin reception and two component signaling among both subspecies. Maintenance of stem cell fate in the SAM is controlled by a regulatory network between *CLAVATA* (*CLV*) ligand-receptor system and the homeodomain protein *WUSCHEL* [*WUS*]^56^. Auxin and Cytokinin also play an important role in *WUSCHEL* mediated maintenance of stem cell niche in SAM^57^. Recently cytokinin mediated *MOC3* (*Os04g0663600*), a WUSCHEL-LIKE HOMEOBOX 1, reported to affect tiller bud formation in rice^58^. Auxin mediated Monopter is an inhibitor of cytokinin mediated type A response regulator, which is a key mediator for maintenance of SAM^59^. Down and Up regulation of *ARF5* and *OsRR3*, respectively in *japonica* compare to *indica* may lead to less differentiation and regeneration in *indica*, thereby indicating the stark contrast between the two subspecies.TDZ mediated overexpression of cytokinin signaling mediated genes has already been reported^10^. Cytokinin also involve in positive regulation of SAM maintenance through Cytokinin-*KNOX* mediated signaling in rice^60^. Type A response regulator act as feedback inhibitor of cytokinin signaling^61^. Down regulation of *OsRR3* and other *OsRRs* in *indica* may lead to imbalance between SAM maintenance and differentiation due to increase activity of cytokinin-*KNOX* signaling which may ultimately responsible for higher cell division but lesser differentiation and regeneration in *indica* compare to *japonica*. FPKM based differential expression pattern of some important meristem maintenance and somatic embryogenesis related genes among *japonica* and *indica* subspecies, described above, may observe in Table S9. Although due to less information about SAM maintenance in rice, further characterization may explore signature genes involve in the whole process in a better way. Although, several reports are available about role of phytohormones including auxin, cytokinin, and BR in regulating somatic embryogenesis and regeneration in monocots including rice but molecular regulation is still unexplored. Differential expression pattern of phytohormone signaling mediated genes may give a detailed dynamic and differential regulation of key regulators involve in somatic embryogenesis in rice. Moreover, differentially expressing genes of individual families between *japonica* and *indica* may be helpful to explore genomics behind contrasting phenotype among both subspecies.

### Expression analysis of SERK family receptor-like protein kinase and DNA methyltransferase genes

**Figure 9:**
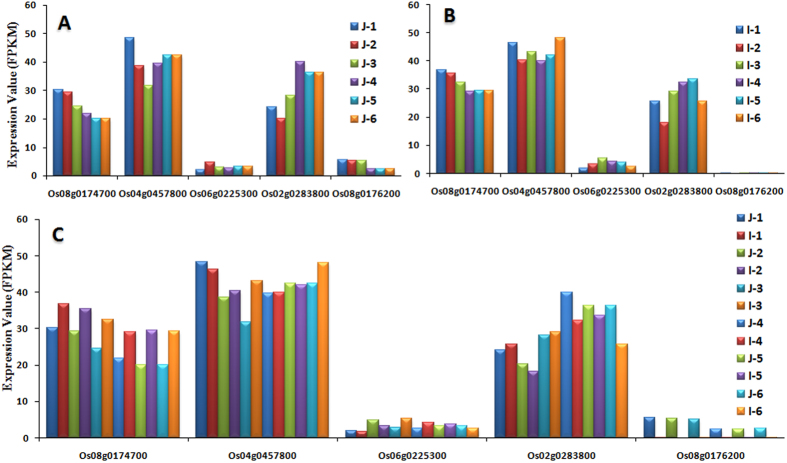
Expression pattern analysis of *Somatic Embryogenesis Responsive Kinase* (*SERK*) gene family of rice showing unique expression of one of the SERK like gene, *Os08g0176200*. (**A**) Dynamic expression pattern in *japonica* subspecies, (**B**) Dynamic expression pattern in *indica* subspecies, (**C**) Differential Expression among both *japonica* and *indica* subspecies of rice. Colors bars showing different stages of both subspecies individually.

Somatic Embryogenesis Receptor Kinases (*SERK*s), a leucine rich repeats containing family and DNA methyltransferases play a vital role in somatic embryo competence and meristem development. To explore possible role of *SERK* gene family in rice, FPKM based dynamic and differential expression analysis was carried out (Fig. 9, Table S10). Five selected members of *SERK* gene family were analyzed for expression analysis. As shown in Fig. 9, among all representative genes of *SERK* family, *OS08G0176200* was found to be expressed in *japonica* sub-species only where as its expression was almost negligible in *indica* (Fig. 9, Table S9). Expression of this gene was higher in initial stages of development. Role of *SERKs* in somatic embryogenesis have been reported in many plants including rice. Initially Yukihiro Ito *et al.*^62^ isolated two *SERK* (*SERK1* and *SERK2*) and showed their expression in some specific tissues^62^. Later on Singla *et al.*^63^ reported structural characterization and expression analysis of the *SERK/SERL* gene family in rice^63^. Differential expression pattern of one of the member of *SERK* family indicating its probable role in initial stages of *in vitro* development. *SERKs* and *SERLs* belong to receptor-like kinase (*LRR*-*RLK*) family^63^. The *leucine-rich repeat* (*LRR*) containing proteins are part of a larger entity called the *NB-ARC* domain as discussed earlier^45^. Therefore, in addition to specific overexpression of *SERKs*, probable role of other differentially expressing *NB-ARC* domain containing genes, described previously, cannot be ignored. Further, DNA methyl transferase mediated developmental regulation in rice was also analyzed and some of the genes showed differential expression pattern among both subspecies (Table S11). Role of cytosine DNA methyltransferases in rice meristem development have already been reported^64^. Differential expression pattern of such genes may be associated with somatic embryogenesis pattern between *japonica* and *indica* subspecies of rice. Detailed analysis and characterization of selected genes described above may provide further molecular signaling for better understanding of the problem in a precise manner.

**Figure 10:**
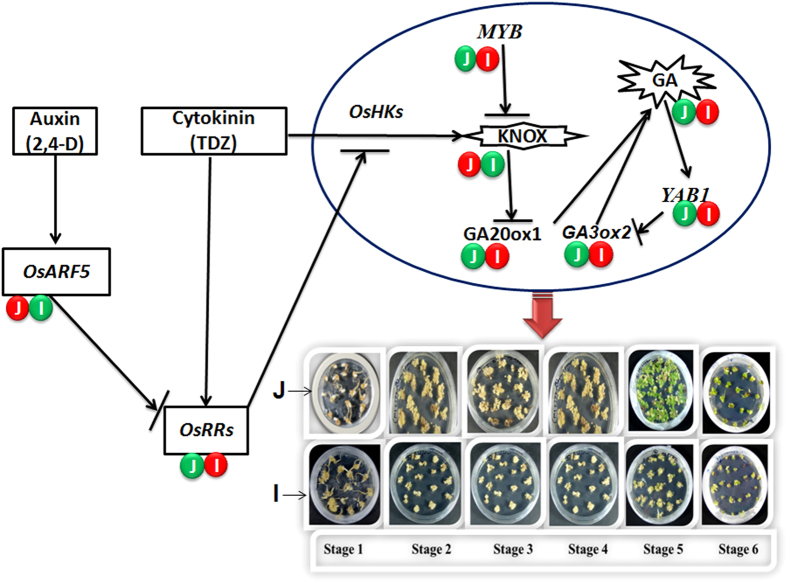
Putative layout showing summary of possible signaling pathway regulating differential somatic embryogenesis and regeneration responses among *japonica* and *indica* subspecies of rice. Down regulation of positive regulators of stem cell maintenance and meristem development (i.e. *KNOX*, *OsARF5*) and up regulation of its counterparts (*OsRRs*, *MYB*, *GA20ox1*/*GA3ox2*) in *japonica* may be responsible for its better regeneration and differentiation of somatic embryos as compared to *indica*. Green and red colored shapes showing up regulated and down regulated genes respectively among *japonica* (J) and *indica* (I) subspecies. Os- RRs, ARF, HKs; Oryza sativa- Response Regulators, Auxin Response Regulators, Histidine Kinases.

Phytohormones play an important role in stem cell maintenance by affecting its key regulators directly or indirectly. *KNOX* acts as a positive regulator of stem cell maintenance through cytokinin-*KNOX* signaling in rice somatic embryogenesis. Auxin responsive factor 5 mediated down regulation of cytokinin Type A response regulators triggers down regulation of *KNOX* through inhibiting cytokinin-*KNOX* signaling in *japonica*. Moreover *KNOX* also down regulated by one of the *MYB* gene in *japonica* (Fig. 10). Product of *KNOX* acts as an inhibitor of GA biosynthesis. Up regulation of GA biosynthetic genes *GA20ox1* and *GA3ox2* due to *KNOX* down regulation in japonica induce increased rate of GA biosynthesis in *japonica* somatic embryogenesis process as compared to *indica*. As a whole, down regulation of positive regulators of stem cell maintenance (i.e. *KNOX*, *OsARF5*) and up regulation of its counterparts (*OsRRs*, *MYB*, *GA20ox1*/*GA3ox2*) in *japonica* may be responsible for its better regeneration and differentiation of somatic embryos as compared to *indica*. Moreover, dynamic and differential expression pattern of meristem regulatory genes further support the intriguing possibility that apical meristem development *in vivo* may also regulate embryogenic meristematic cells *in vitro* (i.e. somatic embryogenesis).

## Materials and Methods

### Tissue culture

Mature, dehusked seeds of the rice subspecies, *Oryza sativa* L. ssp. *japonica* and ssp. *indica* were taken and washed thoroughly with MQ water. Seeds were surface sterilized with 90% alcohol for 90 Sec, then immediate 3–5 times washing with MQ water to remove alcohol followed by 2% NaClO with 1–2 drop Tween 20 for 45 minutes, again 8–10 time washing to remove NaClO. Two different media sets, MS [Murashige and Skoog]^65^ and N6 basal media were used for callus induction. Hundred seeds were pre-cultured in each medium and incubated at 26 °C in the dark. After 3–4 weeks, the proliferating calli were sub cultured onto the same medium and cultured for another 3–4 weeks. For somatic embryogenesis and regeneration, white friable proembryogenic calli were transferred to both N6 and MS regeneration medium with and without TDZ. Basic rice tissue culture protocol was carried out following the method of Chakrabarty *et al.*^10^. Culture media and respective ingredients are described in Table S12.The regeneration efficiency of rice calli was measured as a percentage of callus induction (i.e., number of seeds giving rise to callus/total number of seeds inoculated).

### Plant Material, RNA isolation and quality controls

Callus sample of selected stages with at least three independent sample of each stage were harvested, ground in liquid N_2_ and stored at −80 °C. Frozen tissues were ground to a fine powder in liquid nitrogen and total RNA was extracted using RNeasy plant Mini Kit (QIAGEN, MD) and treated with RNase free DNaseI (QIAGEN, MD) according to manufacturer’s instructions. The quality and quantity of total RNA were analyzed by agarose gel and spectrophotometric analysis (ND1000 Nanodrop, NanoDrop Technologies, USA). Quantity as well as quality of pooled RNA was again checked using Agilent 2100 Bioanalyzer RNA chip (Agilent Technologies Inc., Santa Clara, CA). Only the RNA samples with 260 of 280 ratios from 1.8 to 1.9, 260 of 230 ratios from 2.0 to 2.5 and RIN (RNA integrity number) more than 9.0 were used for the analysis. The equal amount of total RNA from the three independent samples of each stage were pooled and used for further processing.

### Illumina sequencing

The cDNA libraries were generated using mRNA assay for sequencing on Illumina HiSeq 2000 sequencing platform. Paired-end cDNA library was generated from all samples and sequencing was performed to generate the ~101bp paired-end reads. Quality controls and adaptor removal was done by NGSQCTOOLKIT (http://www.nipgr.res.in/ngsqctoolkit.html) software^66^. This software was used for filtering of high quality reads based on quality score (Q > 30). Based on quality of sequence reads, we trimmed sequence read where necessary using NGSQCTOOLKIT; to retain only high quality sequence for further analysis. In addition, the low-quality sequence reads were excluded from the analysis. We got ~85% high quality reads in each library from generated data.

### Read Mapping and Gene Expression Analysis

The pre-processed reads were aligned to the reference rice genome and gene model downloaded from Ensembl (http://plants.ensembl.org/Oryza_sativa/Info/Index; Genome version = Oryza_sativa.MSU7.21.genome.fa). The alignment was performed using Tophat program version 2.0.8 (http://ccb.jhu.edu/software/tophat/index.shtml) with default parameters^67^. We only used mapped reads for further analysis. We calculated the number of uniquely mapped reads for each gene model in the Oryza_sativa.MSU7.21.genome.fa^68^. The aligned reads were used for estimating expression of the genes and transcripts using cufflinks program (version 2.0.2). FGS were identified by parsing the alignment output files from Tophat, and then normalized the resulting read counts by FPKM to measure the gene expression level. Differential expression analysis was performed using cuffdiff program [version 2.0.2]^69^ using cufflink package (http://cole-trapnell-lab.github.io/cufflinks/manual/).

Tophat mapping for individual samples of both subspecies was also performed with their respective CDS database (ftp://ftp.ensemblgenomes.org/pub/plants/release30/fasta/oryza_ sativa/cds/ and ftp://ftp.ensemblgenomes.org/pub/plants/release-30/fasta/oryza_indica/cds/) to analyze unique transcript abundance among both subspecies. Mapped genes from alignment files were extracted and used as raw files for stage specific reciprocal blast analysis to find out unique transcripts among both subspecies. Expression pattern of unique transcripts of *japonica* and *indica* subspecies was analyzed using their respective gene model downloaded from Ensembl and cufflink package as mentioned previously.

### PCA and Hierarchical Clustering

To facilitate graphical interpretation of relatedness among 12 different samples, we reduced the dimensional expression data to two dimensions by PCA using the FactoMineRpackage in R software with default settings [https://cran.r-project.org/web/packages/FactoMineR/index.html^70^. Hierarchical clustering was performed by the pvclust package (https://cran.r-project.org/web/packages/pvclust/index.html) with default settings using Pearson’s correlation coefficient^71^. The normalized gene expression values in terms of FPKM were used for the analysis of PCA and hierarchical clustering.

### Functional and differential gene expression analysis

To retrieve the detailed GO annotation, all the up and down regulated genes in *japonica* with respect to *indica* having fold change (≤−1 and ≥1) and p-value ≤ 0.05 were analyzed using agriGo online tool (http://bioinfo.cau.edu.cn/agriGO/analysis.php) following Singular Enrichment Statistical analysis^72^. The heatmap of the annotated pathways, was generated using PageMan^73^ with average statistics type and Banjamini Hochberg multiple testing correction for all the differentially regulated genes in various developmental stages of *japonica* with fold change (≤−1 and ≥1) and p-value ≤ 0.05.

### Cluster analysis

All the genes were analyzed in MeV 4.2 (http://sourceforge.net/projects/mev-tm4/files/mev-tm4/) to perform KMC cluster analysis for both *japonica* and *indica* sub-species separately using expression values in terms of FPKM. Fifty clusters were generated on the basis on Euclidean Distance Metric. To generate the heatmap of corresponding pathways, cluster number 1, 6, 20, 25 and cluster number 1, 3, 7, 10 were selected from *japonica* and *indica*, respectively. Raw gene files of selected clusters were analyzed in PageMan^73^ using average statistics type and Banjamini Hochberg multiple testing correction.

### Expression analysis using qRT PCR

Real time PCR was performed in 20 μl for a set of selected genes using Power SYBR Green PCR Master Mix (ABI, USA). The list of selected genes and oligonucleotide primers (Eurofins, India) used for each gene are listed in Table S13. Oligonucleotide primers for rice actin gene (Table S13) were used as the internal control for establishing equal amounts of cDNA in all reactions. The reactions were performed using the following cycle conditions, an initial 94 °C for 2 min, followed by 30 cycles of 94 °C for 30 s, 60 °C for 30 s, and 72 °C for 30 s, and the final 5 min extension at 72 °C. The qPCR data was analysed with the delta-delta CT method using Actin as reference gene^74^. All the experiments were repeated using three biological replicates and the data were analyzed statistically (±Standard Deviation).

### Data access

The Illumina sequencing reads of 6 samples of *japonica* and *indica* have been submitted to NCBI (http://www.ncbi.nlm.nih.gov/) as SRA296392 and SRA296393, respectively. Raw file containing FPKM values of all samples is available in Table S14.

## Additional Information

**How to cite this article**: Indoliya, Y. *et al.* Decoding regulatory landscape of somatic embryogenesis reveals differential regulatory networks between *japonica* and *indica* rice subspecies. *Sci. Rep.* **6**, 23050; doi: 10.1038/srep23050 (2016).

## Supplementary Material

Supplementary Information

Supplementary Table S2A-B

Supplementary Table S4

Supplementary Table S5

Supplementary Table S6

Supplementary Table S8

Supplementary Table S9

Supplementary Table S11

Supplementary Table S14

## Figures and Tables

**Table 1:** Summary of the fastq file generated from RNA sequencing showing average statistics of all samples individually.

Sample ID	Total Reads	Total data (Gb)	% GC	% of data > = 30 Phred Score	Number of aligned reads to reference genome (%)
J 1	13,15,87,518	13.29	49.8	88.89	122,381,206(93)
I 1	14,91,87,546	15.06	50.99	88.64	132,882,776(89.07)
J 2	6,13,73,804	6.19	49.07	90.74	58,178,890(94.79)
I 2	5,55,18,136	5.6	49.45	90.56	50,626,532(91.19)
J 3	4,95,90,822	5	49.2	90.78	46,923,064(94.62)
I 3	5,37,82,624	5.43	48.67	90.7	48,993,232(91.10)
J 4	6,50,18,220	6.56	50.28	90.99	61,516,573(94.62)
I 4	7,82,64,970	7.9	49.92	91.1	68,955,396(88.11)
J 5	8,19,71,282	8.27	50.44	80.16	69,393,745(84.67)
I 5	4,68,34,256	4.73	49.24	90.67	42,614,049(90.99)
J 6	5,74,89,266	5.8	49.81	91.1	54,148,321(94.19)
I 6	6,44,00,064	6.5	49.65	91.15	57,864,720(89.85)
