# Supplementary material for: Decoding regulatory landscape of somatic embryogenesis reveals differential regulatory networks between japonica and indica rice subspecies
Source: Sci Rep. 2016 Mar 14;6:23050. doi: 10.1038/srep23050 (PMC4789791; doi:10.1038/srep23050)
Supplement: Supplementary Information [file srep23050-s1.pdf]

**Decoding regulatory landscape of somatic embryogenesis reveals differential regulatory networks between *japonica* and *indica* rice subspecies**

Yuvraj Indoliya[1,2], Poonam Tiwari[1], Abhisekh Singh Chauhan[1], Ridhi Goel[1,2], , Manju Shri[1], Sumit Kumar Bag[1,2], Debasis Chakrabarty[1,2*]

[1] Council of Scientific and Industrial Research - National Botanical Research Institute (CSIR-NBRI), Rana Pratap Marg, Lucknow-226001, India

[2] Academy of Scientific and Innovative Research (AcSIR), Anusandhan Bhawan, 2 Rafi Marg, New Delhi-110 001, India

*** Corresponding author chakrabartyd@nbri.res.in**

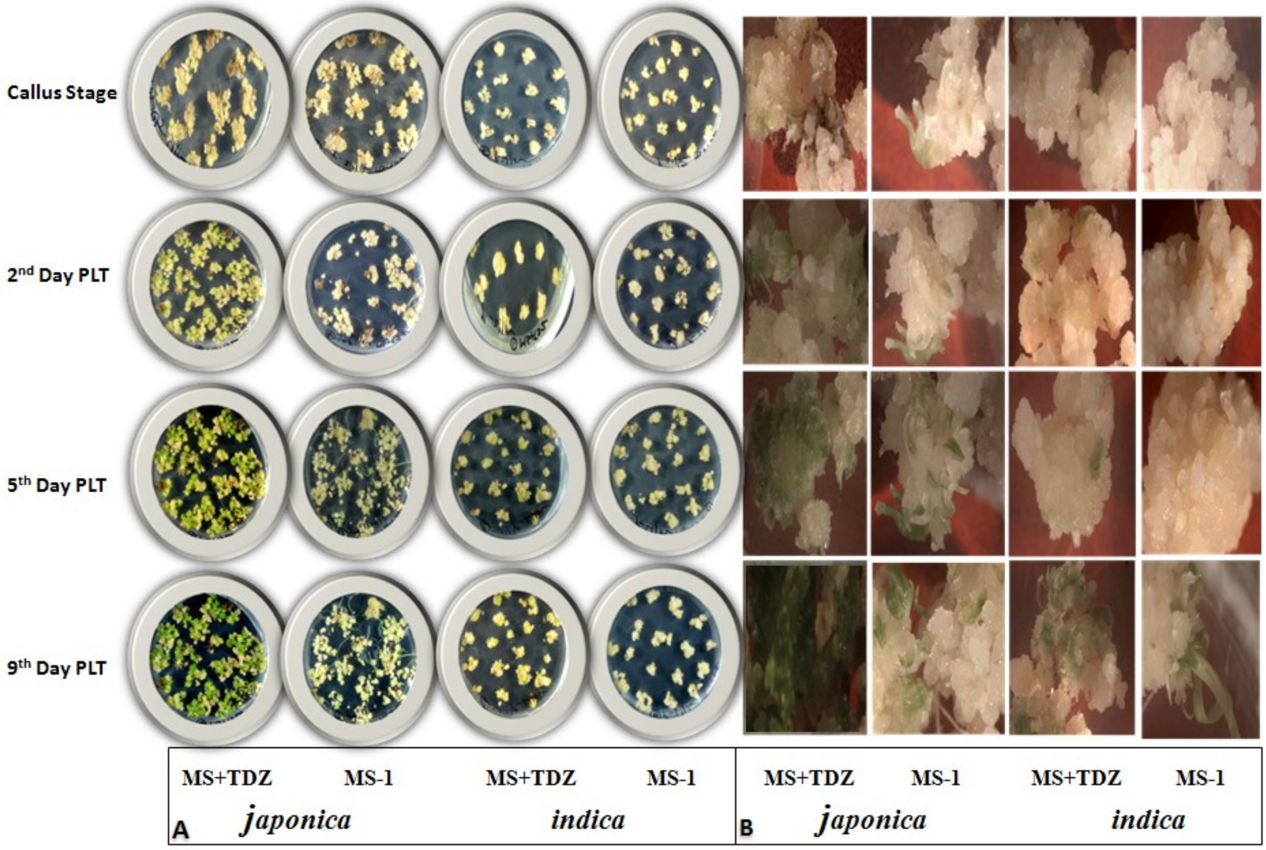


**Figure S1 |** Comparative somatic embryogenesis and regeneration frequency in MS+TDZ and MS-1 (without TDZ) regeneration medium among both *japonica* and *indica* subspecies showing high efficiency in *japonica* subspecies of rice compare to *indica*.

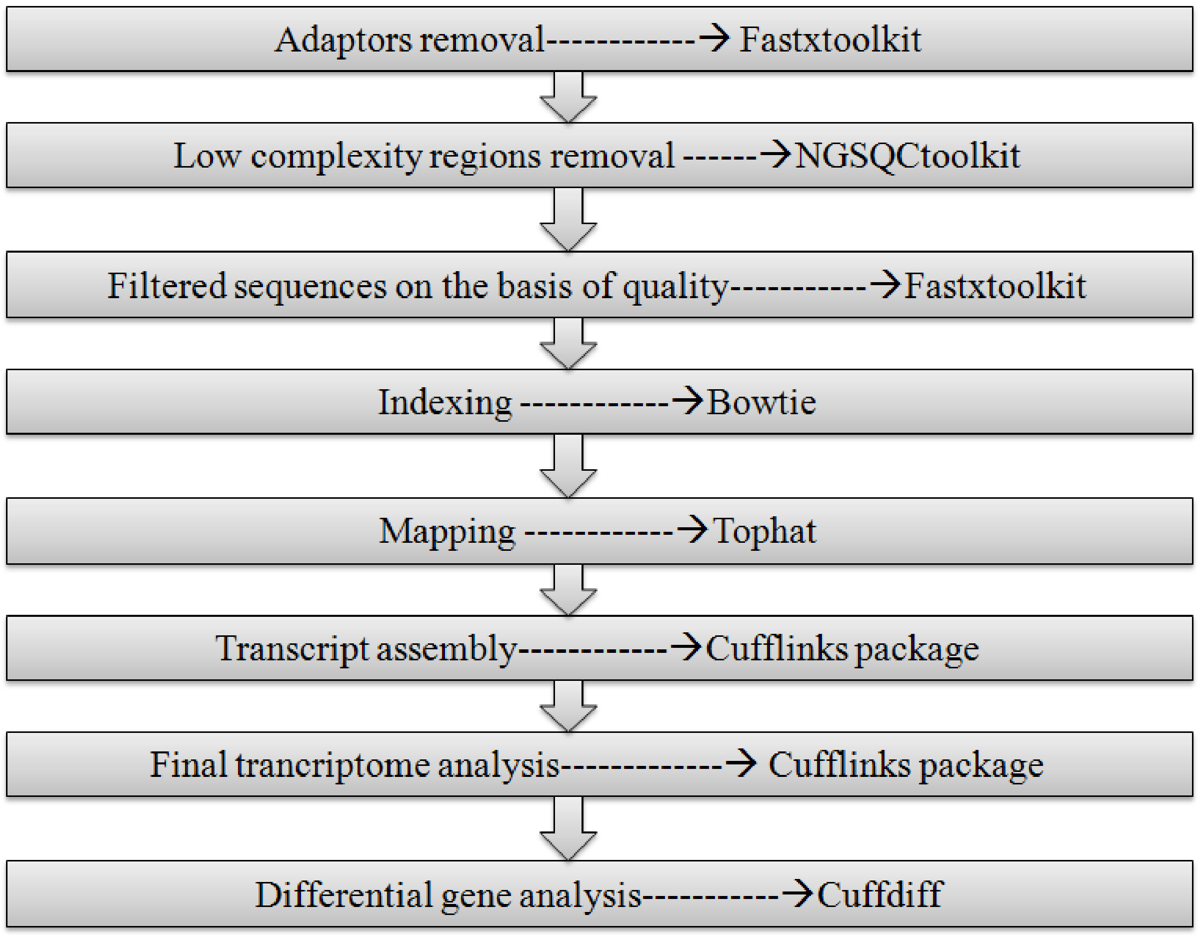


**Figure S2 |** Sequential steps of transcriptome analysis with respective tools used in the study.

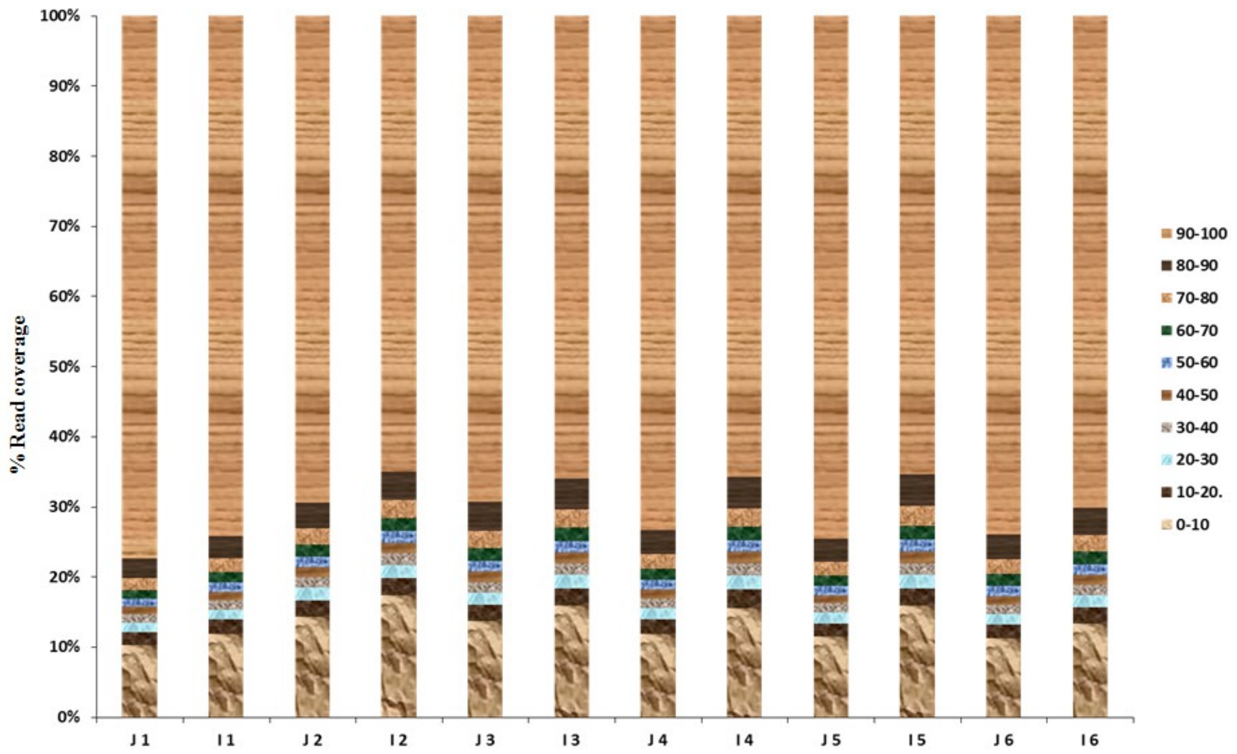


**Figure S3 |** Dynamic and differential transcriptome relationships based on percentage read coverage in different time points of *japonica* and *indica* subspecies showing maximum percentage of read coverage between 90-100.

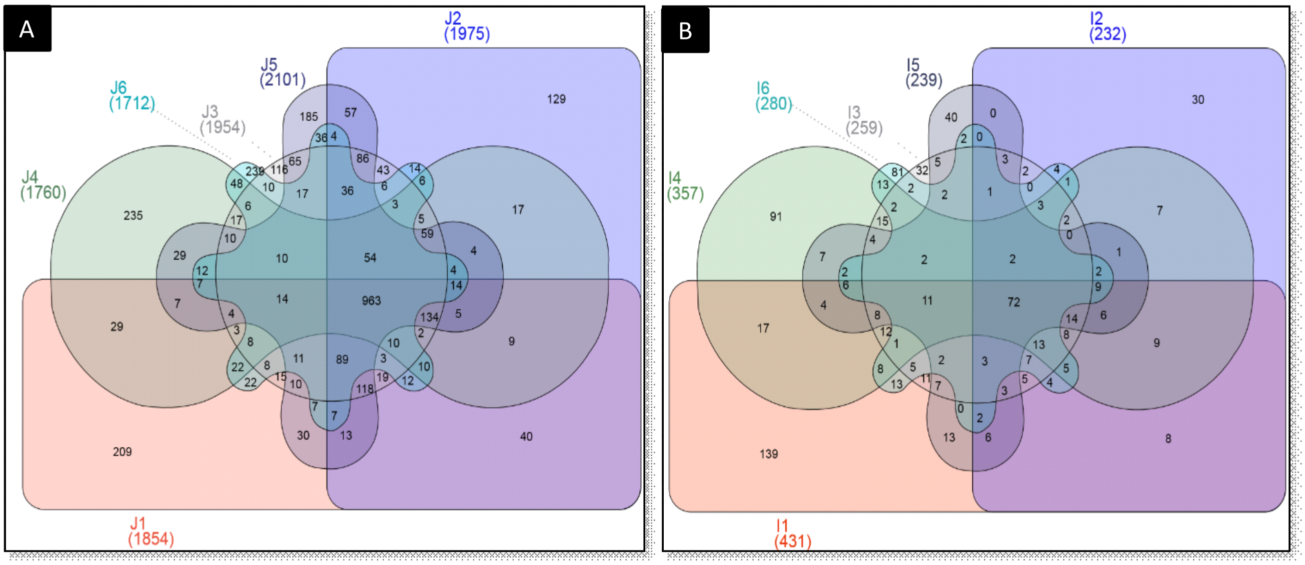


**Figure S4 |** Analysis of unique genes expressed among *japonica* and *indica* subspecies. A) Unique gene of *japonica* compare to *indica*; B) Unique gene of *indica* compare to *japonica.* Different color codes represent individual stages.

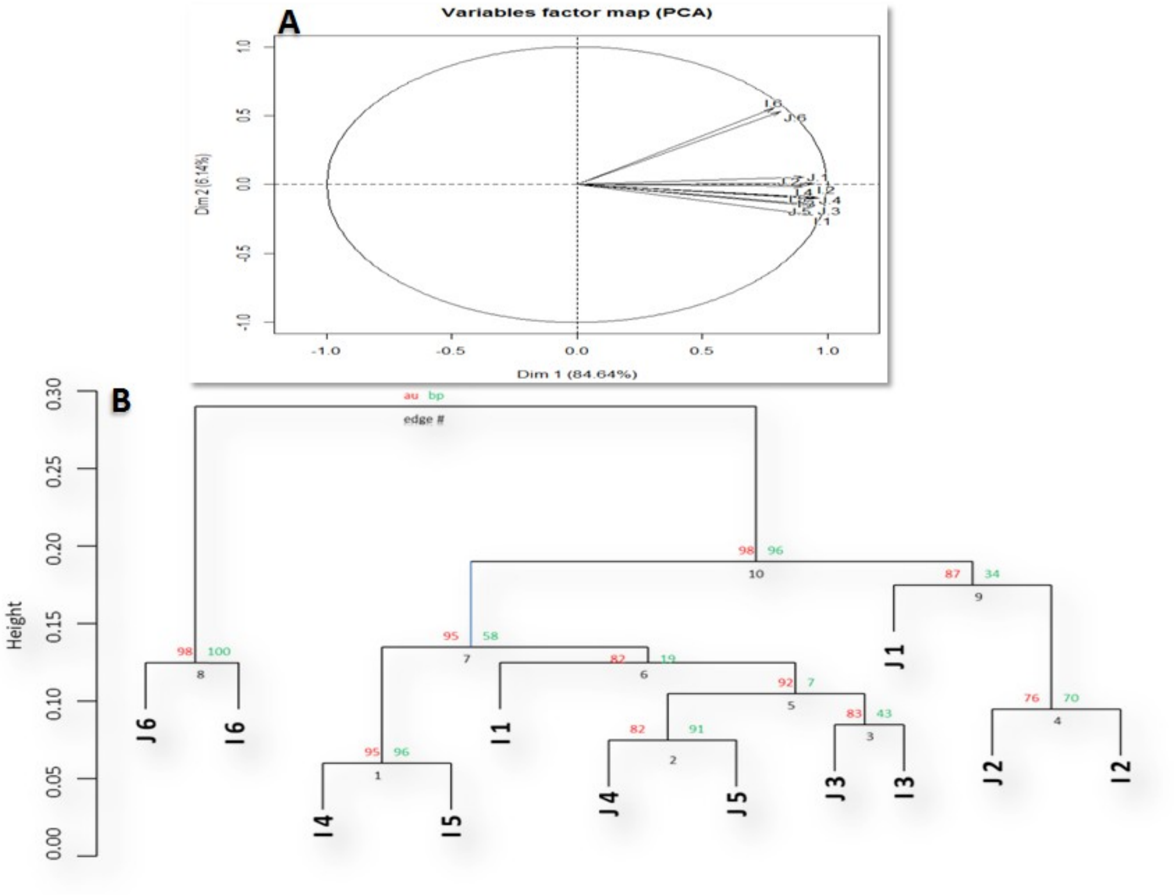


**Figure S5 |** Global transcriptome relationships among different samples. A) PCA of the RNA-seq data for the 12 samples shows 4 distinct groups. B) Cluster dendogram showing global transcriptome relationships among time series samples between *japonica* and *indica.*

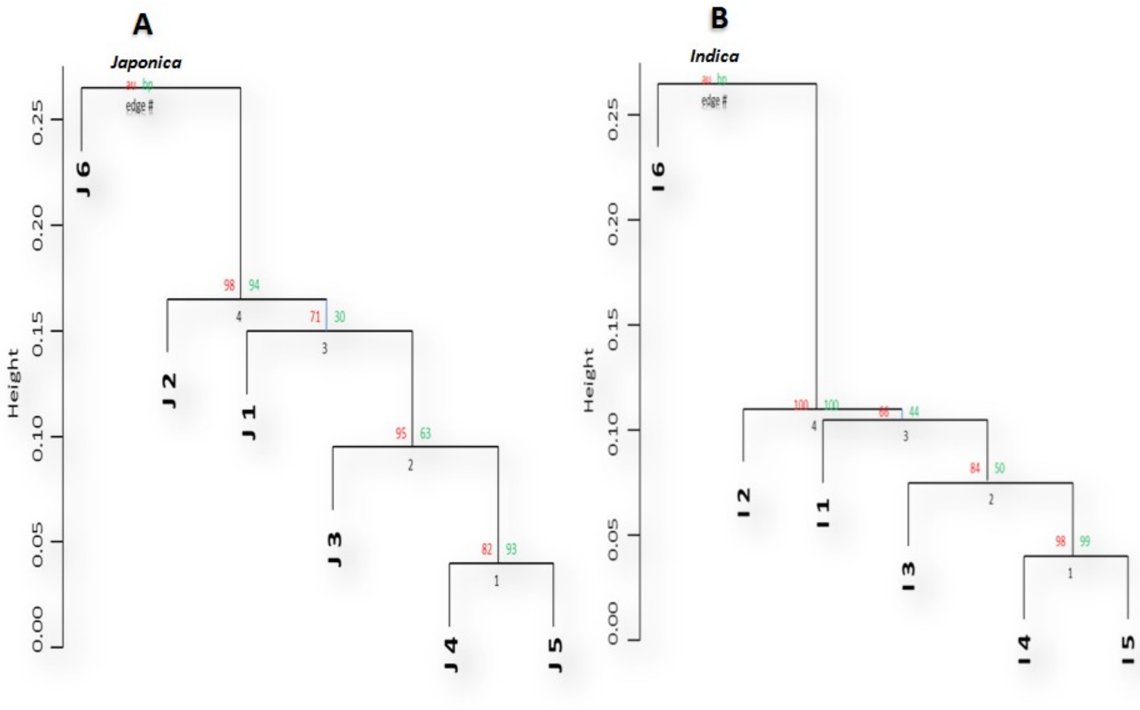


**Figure S6 |** Global transcriptome relationships among different samples. Cluster dendogram showing global dynamic transcriptome relationships among time series samples of *japonica* (A) and i*ndica* (B).

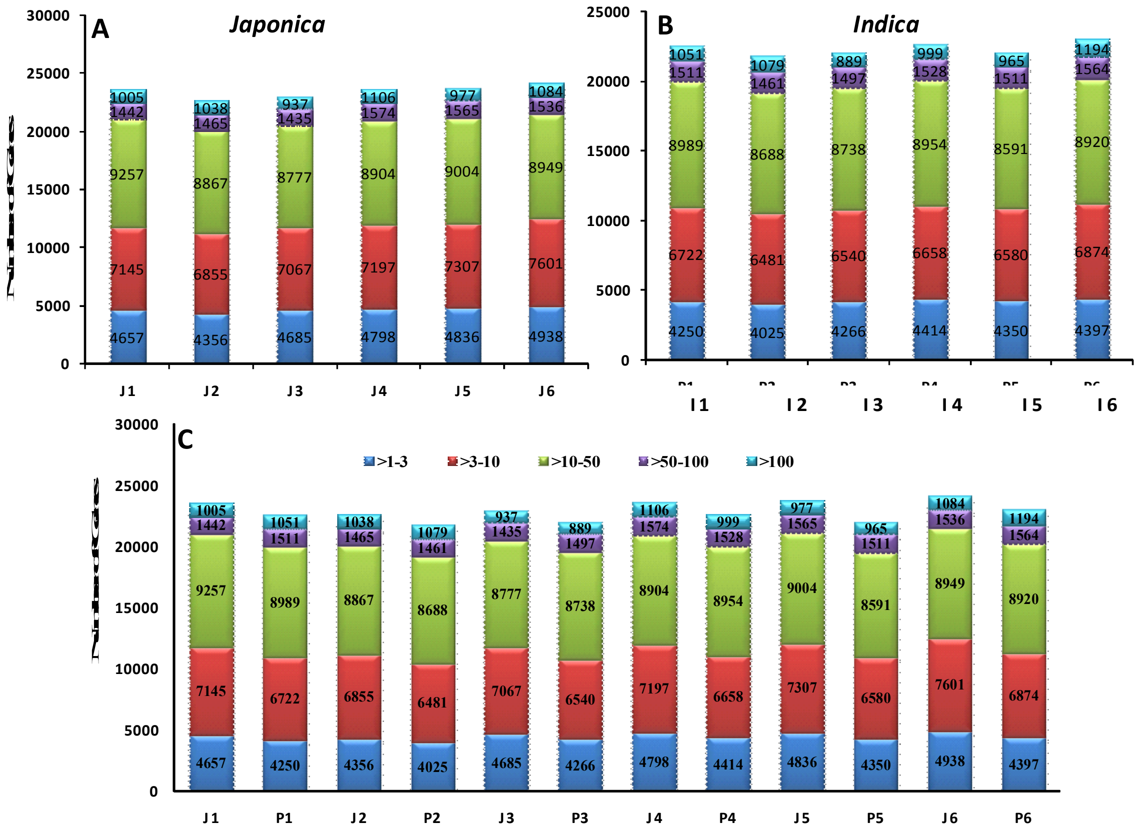


**Figure S7 | FPKM based** dynamic and differential expression analysis of the rice transcriptome. A) Dynamics pattern in *japonica* subspecies, B) Dynamic pattern in *indica* subspecies, C) Differential expression pattern among both subspecies Number of transcripts with different expression abundances in various tissue samples based on the FPKM values showing highest and lowest number of genes lying between >10-50 and >100 FPKM respectively. The transcripts showing FPKM values of 1 to 3, greater than 3 to 10, greater than 10 to 50, greater than 50 to 100, and greater than 100 have been classified as very lowly, lowly, moderately, highly, and very highly expressed, respectively.

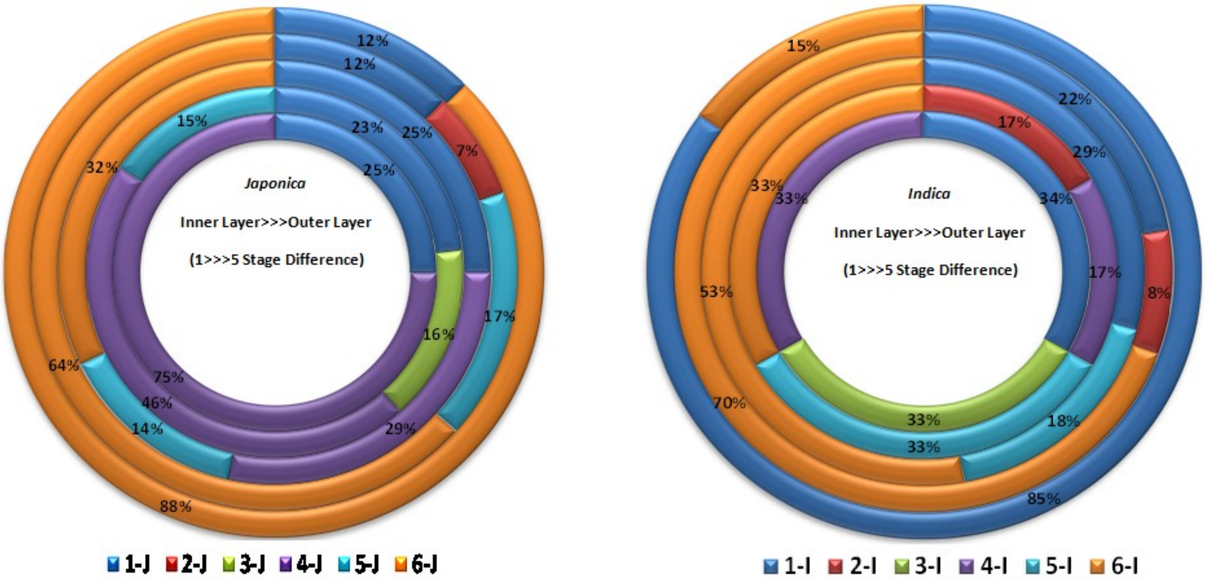


**Figure S8 |** Number of transcripts showing specific expression with different expression abundances in various stages of *japonica* and *indica*. The stage specific transcripts are represented by three or greater fold change in the sample of interest compared to other. Individual stages of *japonica* and *indica* indicated by respective colors.

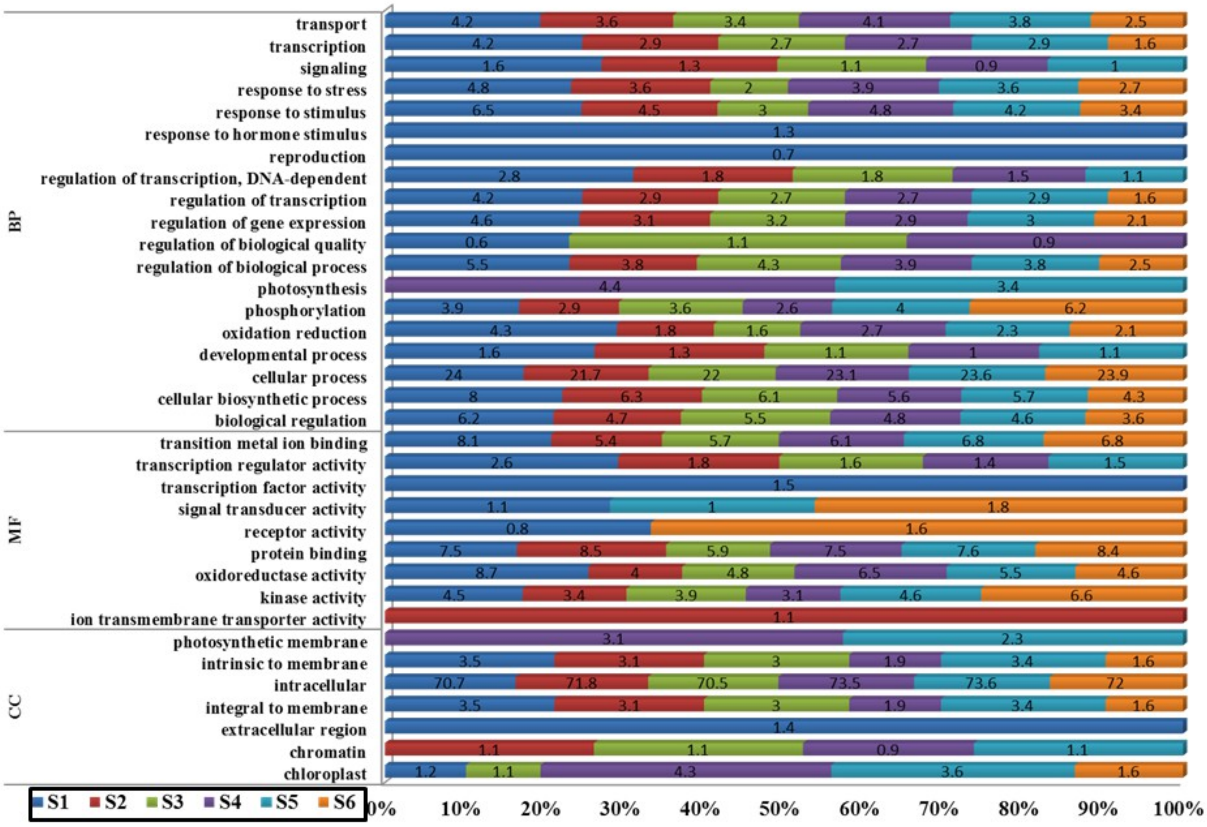


**Figure S9 |** Gene annotation and functional categorization of differentially expressing genes (DEGs) using AgriGO singular enrichment analysis tool showing percentage of differential genes in respective stages. BP: Biological Process, CC: Cellular Component, MF: Molecular Function. Different color bars showing respective stages.

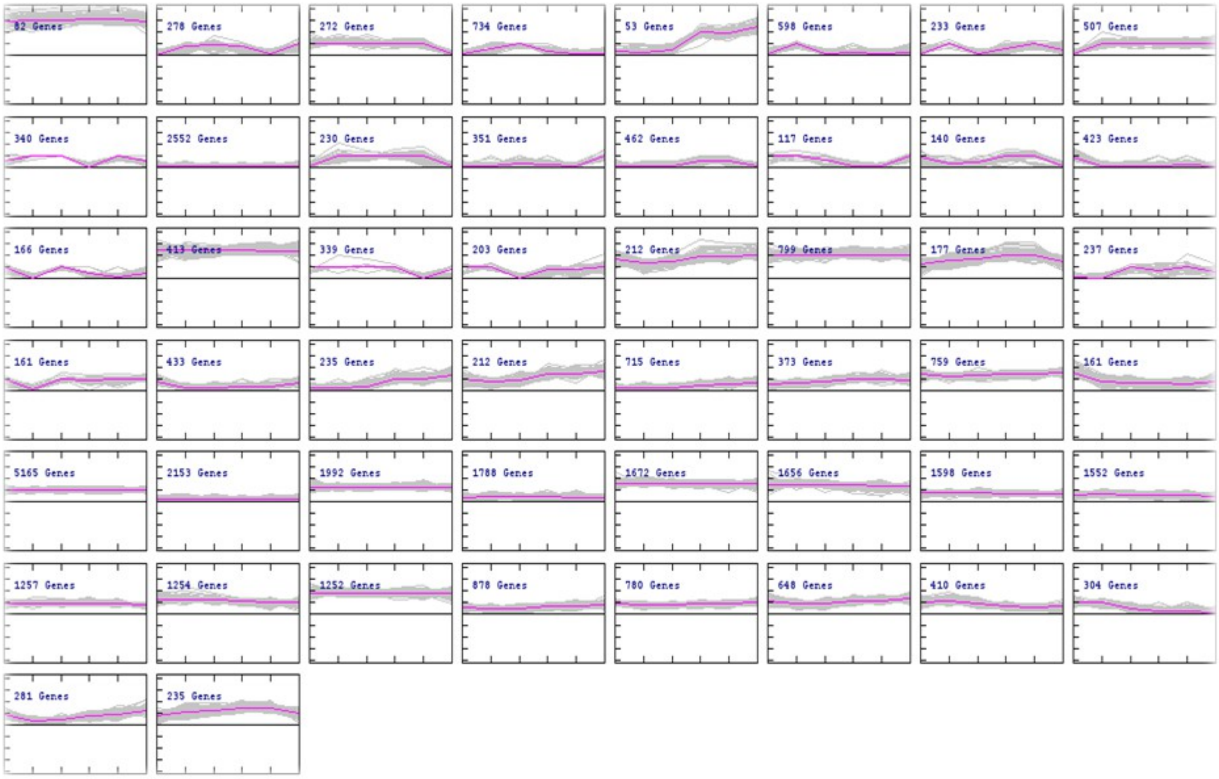


**Figure S10A |** Clusters based expression pattern analysis of *japonica* showing raw file of all 50 clusters prepared using 'Euclidean Distance Metrics' (See materials and methods section for further information).

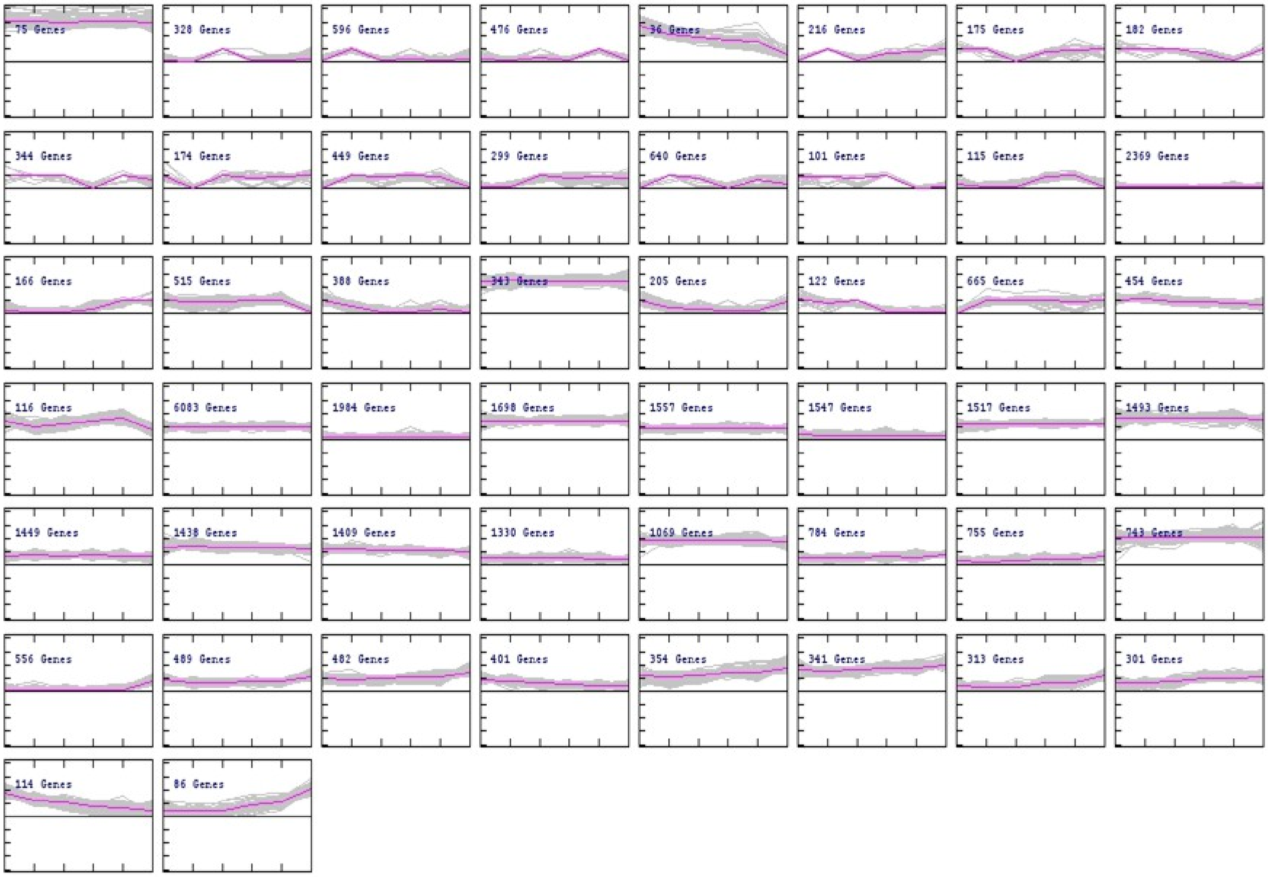


**Figure S10B |** Clusters based expression pattern analysis of *indica* showing raw file of all 50 clusters prepared using 'Euclidean Distance Metrics' (See materials and methods section for further information).

**Table S1 | Proembryogenic calli regeneration statistics after 9 days dark treatment. Green blocks showing early meristem differentiation start point (Morphologically Visible)**

| Number of Days | S .No. | *japonica* | | *indica* | |
|---|---|---|---|---|---|
| | | MS+TDZ | MS | MS+TDZ | MS |
| | 1 | 0 | 0 | 0 | 0 |
| | 2 | 0 | 0 | 0 | 0 |
| | 3 | 0 | 0 | 0 | 0 |
| | 4 | 0 | 0 | 0 | 0 |
| 0 Day | 5 | 0 | 0 | 0 | 0 |
| | 6 | 0 | 0 | 0 | 0 |
| | 7 | 0 | 0 | 0 | 0 |
| | 8 | 0 | 0 | 0 | 0 |
| | 1 | 8 | 1 | 1 | 0 |
| | 2 | 15 | 3 | 4 | 0 |
| | 3 | 7 | 3 | 0 | 0 |
| | 4 | 38 | 4 | 0 | 0 |
| | 5 | 10 | 3 | 0 | 0 |
| | 6 | 24 | 3 | 5 | 0 |
| 1st Day PLT | 7 | 20 | 7 | 1 | 0 |
| | 8 | 28 | 4 | 3 | 0 |
| | 1 | 20 | 3 | 3 | 0 |
| | 2 | 35 | 4 | 8 | 0 |
| | 3 | 15 | 5 | 0 | 0 |
| | 4 | 40 | 8 | 0 | 0 |
| | 5 | 17 | 9 | 0 | 0 |
| | 6 | 33 | 12 | 10 | 0 |
| 2nd Day PLT | 7 | 29 | 11 | 2 | 0 |
| | 8 | 40 | 6 | 3 | 0 |
| | 1 | 22 | 5 | 5 | 0 |
| 3rd Day PLT | 2 | 35 | 6 | 15 | 0 |
| | 3 | 16 | 5 | 1 | 0 |
| | 4 | 40 | 8 | 1 | 0 |
| | 5 | 20 | 9 | 1 | 0 |
| | 6 | 34 | 12 | 13 | 0 |
| | 7 | 29 | 15 | 8 | 0 |
| 3rd Day PLT | 8 | 40 | 10 | 7 | 0 |
| | 1 | 70 | 10 | 6 | 1 |
| | 2 | 80 | 9 | 20 | 0 |
| | 3 | 59 | 7 | 4 | 0 |
| | 4 | 60 | 12 | 4 | 0 |
| | 5 | 53 | 11 | 2 | 0 |
| | 6 | 68 | 12 | 20 | 0 |
| 4th Day PLT | 7 | 64 | 20 | 13 | 0 |
| | 8 | 72 | 14 | 10 | 0 |
| | 1 | 75 | 15 | 8 | 1 |
| | 2 | 89 | 10 | 22 | 0 |
| | 3 | 78 | 12 | 5 | 0 |
| | 4 | 95 | 14 | 7 | 0 |
| | 5 | 69 | 16 | 3 | 2 |
| | 6 | 80 | 12 | 22 | 0 |
| 5th Day PLT | 7 | 73 | 22 | 16 | 1 |
| | 8 | 98 | 15 | 12 | 1 |
| | 1 | 80 | 16 | 9 | 1 |
| | 2 | 110 | 13 | 22 | 0 |
| | 3 | 90 | 15 | 6 | 0 |
| | 4 | 130 | 16 | 7 | 0 |

| | | | | | |
|---|---|---|---|---|---|
| 6th Day PLT | 5 | 80 | 13 | 4 | 2 |
| | 6 | 110 | 14 | 25 | 0 |
| | 7 | 90 | 23 | 16 | 2 |
| | 8 | 120 | 19 | 12 | 1 |
| 7th Day PLT | 1 | 80 | 18 | 9 | 1 |
| | 2 | 110 | 15 | 22 | 0 |
| | 3 | 90 | 19 | 6 | 0 |
| | 4 | 130 | 18 | 7 | 0 |
| | 5 | 80 | 14 | 4 | 3 |
| 7th Day PLT | 6 | 110 | 15 | 25 | 0 |
| | 7 | 90 | 25 | 16 | 2 |
| | 8 | 120 | 21 | 12 | 1 |
| 8th Day PLT | 1 | 80 | 20 | 9 | 1 |
| | 2 | 110 | 18 | 22 | 0 |
| | 3 | 90 | 20 | 6 | 0 |
| | 4 | 130 | 20 | 7 | 0 |
| | 5 | 80 | 16 | 4 | 3 |
| | 6 | 110 | 17 | 25 | 0 |
| | 7 | 90 | 25 | 16 | 2 |
| | 8 | 120 | 22 | 12 | 1 |
| 9th Day PLT | 1 | 80 | 20 | 9 | 1 |
| | 2 | 110 | 18 | 22 | 0 |
| | 3 | 90 | 20 | 6 | 0 |
| | 4 | 130 | 20 | 7 | 0 |
| | 5 | 80 | 16 | 4 | 3 |
| | 6 | 110 | 17 | 25 | 0 |
| | 7 | 90 | 25 | 16 | 2 |
| | 8 | 120 | 22 | 12 | 1 |
| 0 Day | 1 | (26) 0 | (25) 0 | (25) 0 | (20) 0 |
| | 2 | (25) 0 | (24) 0 | (24) 0 | (24) 0 |
| | 3 | (20) 0 | (24) 0 | (30) 0 | (23) 0 |
| 1st Day | 1 | (26) 4 | (25) 0 | (25) 1 | (20) 0 |
| | 2 | (25) 7 | (24) 0 | (24) 0 | (24) 0 |
| | 3 | (20) 15 | (24) 2 | (30) 0 | (23) 0 |
| 2nd Day | 1 | (26) 18 | (25) 0 | (25) 3 | (20) 0 |
| | 2 | (25) 22 | (24) 3 | (24) 0 | (24) 0 |
| | 3 | (20) 40 | (24) 9 | (30) 0 | (23) 0 |
| 3rd Day | 1 | (26) 30 | (25) 3 | (25) 8 | (20) 0 |
| | 2 | (25) 28 | (24) 8 | (24) 2 | (24) 0 |
| | 3 | (20) 55 | (24) 15 | (30) 1 | (23) 0 |

**Table S3A | Number of genes showing specific expression with different expression abundances in various stages of *japonica*. The stage specific transcripts are represented by three or greater fold change in the sample of interest compare to other**

| Stage Differences | J1 | J2 | J3 | J4 | J5 | J6 |
|---|---|---|---|---|---|---|
| 1 Stage Difference | 1 | 0 | 0 | 3 | 0 | 0 |
| 2 Stage Difference | 3 | 0 | 2 | 6 | 2 | 0 |
| 3 Stage Difference | 7 | 0 | 0 | 8 | 4 | 9 |
| 4 Stage Difference | 5 | 3 | 0 | 0 | 7 | 26 |
| 5 Stage Difference | 2 | 0 | 0 | 0 | 0 | 14 |

**Table S3B | Number of genes showing specific expression with different expression abundances in various stages of *indica*. The stage specific transcripts are represented by three or greater fold change in the sample of interest compare to other**

| Stage Differences | I1 | I2 | I3 | I4 | I5 | I6 |
|---|---|---|---|---|---|---|
| 1 Stage Difference | 2 | 0 | 2 | 2 | 0 | 0 |
| 2 Stage Difference | 0 | 1 | 0 | 1 | 2 | 2 |
| 3 Stage Difference | 5 | 0 | 0 | 0 | 3 | 9 |
| 4 Stage Difference | 8 | 3 | 0 | 0 | 0 | 25 |
| 5 Stage Difference | 17 | 0 | 0 | 0 | 0 | 3 |

**Table S3C | Number of genes showing specific expression with different expression abundances in various stages among *japonica* and *indica*. The stage specific transcripts are represented by three or greater fold change in the sample of interest compare to other**

| Stage Differences | J1 | I1 | J2 | I2 | 3J | 3I | 4J | 4I | 5J | 5I | 6J | 6I |
|---|---|---|---|---|---|---|---|---|---|---|---|---|
| Same Stage Difference | 83 | 17 | 106 | 18 | 116 | 29 | 100 | 25 | 151 | 23 | 70 | 4 |
| 1 Stage Difference | 108 | 21 | 206 | 40 | 199 | 45 | 281 | 49 | 186 | 33 | 118 | 20 |
| 2 Stage Difference | 129 | 33 | 93 | 28 | 217 | 40 | 230 | 22 | 142 | 31 | 82 | 21 |
| 3 Stage Difference | 101 | 40 | 134 | 23 | 77 | 8 | 101 | 18 | 146 | 20 | 124 | 31 |
| 4 Stage Difference | 143 | 36 | 83 | 13 | 0 | 0 | 0 | 0 | 100 | 20 | 135 | 26 |
| 5 Stage Difference | 85 | 30 | 0 | 0 | 0 | 0 | 0 | 0 | 0 | 0 | 79 | 20 |

**Table S7 | FPKM based expression pattern of *cullin* and *LRR* genes among *japonica* and *indica* subspecies of rice**

| **Family** | ***cullin* repeat like domain** | **Leucine rich repeats (*lrr*) family** | | | |
|---|---|---|---|---|---|
| **GENE** | **OS05G0369900** | **OS10G0358200** | **OS12G0500500** | **OS11G0673900** | **OS11G0605100** |
| **J1** | 1.42071 | 1.24286 | 5.74706 | 0.886203 | 5.61312 |
| **I1** | 0.0204375 | 0.005676 | 0 | 0.051366 | 0 |
| **J2** | 1.07441 | 0.121883 | 1.07924 | 0.634167 | 2.92619 |
| **I2** | 0 | 0 | 0 | 0 | 0 |
| **J3** | 1.41078 | 0.014788 | 1.03977 | 0.518582 | 5.41451 |
| **I3** | 0.0249336 | 0.013839 | 0 | 0 | 0 |
| **J4** | 4.40597 | 0.03711 | 0.772539 | 0.700162 | 16.2196 |
| **I4** | 0.0779792 | 0 | 0 | 0 | 0 |
| **J5** | 2.6513 | 0.009996 | 0.416346 | 0.644809 | 25.6788 |
| **I5** | 0.150541 | 0 | 0 | 0 | 0 |
| **J6** | 5.07589 | 0.012475 | 1.37521 | 0.649168 | 16.5655 |
| **I6** | 0.0441294 | 0.012255 | 0 | 0.013863 | 0 |

## Table S10 | Expression pattern of members of SERK gene family between *japonica* and *indica* subspecies of rice

| **Gene** | **J-1** | **I-1** | **J-2** | **I-2** | **J-3** | **I-3** | **J-4** | **I-4** | **J-5** | **I-5** | **J-6** | **I-6** |
|---|---|---|---|---|---|---|---|---|---|---|---|---|
| **OS08G0174700** | 30.1723 | 36.8315 | 29.3834 | 35.4049 | 24.5169 | 32.3438 | 21.8322 | 29.0409 | 20.0024 | 29.4338 | 20.0024 | 29.39 |
| **OS04G0457800** | 48.332 | 46.3661 | 38.5178 | 40.2743 | 31.7606 | 43.0285 | 39.5868 | 39.8848 | 42.4028 | 41.8556 | 42.4028 | 48.1524 |
| **OS06G0225300** | 1.98139 | 1.78069 | 4.78687 | 3.31784 | 2.84024 | 5.40677 | 2.68142 | 4.19194 | 3.18502 | 3.8065 | 3.18502 | 2.56403 |
| **OS02G0283800** | 24.0293 | 25.7177 | 20.1298 | 18.1019 | 28.0775 | 29.1281 | 39.944 | 32.2495 | 36.2978 | 33.5849 | 36.2978 | 25.6461 |
| **OS08G0176200** | 5.55426 | 0.022031 | 5.36476 | 0 | 5.1647 | 0.080644 | 2.34034 | 0.041903 | 2.27561 | 0.064927 | 2.51794 | 0.047571 |

## Table S12 | Media composition of different media sets used in the study

| S.No. | Media | Composition |
|---|---|---|
| **1.** | N6 induction Medium | N6 salts and vitamins, 0.3 g/l casein hydrolysate, 0.5 g/l proline, 3 mg/l 2, 4-D, 30 g/l sucrose, 4 g/l phytagel, pH 5.8 |
| **2.** | MS induction medium | MS salts and vitamins, 0.3 g/l casein hydrolysate, 0.5 g/l proline, 3 mg/l 2, 4-D, 30 g/l maltose, 4 g/l phytagel, pH 5.8 |
| **3.** | MS regeneration medium (With TDZ) | MS salts and vitamins, 0.3 g/l casein hydrolysate, 30 g/l maltose, 1.5 mg/l NAA, 3 mg/l BAP, 1 mg/l TDZ, 4 g/l phytagel, pH 5.8 |
| **4.** | MS regeneration medium (Without TDZ) | MS salts and vitamins, 0.3 g/l casein hydrolysate, 30 g/l maltose, 1.5 mg/l NAA, 3 mg/l BAP, 4 g/l phytagel, pH 5.8 |
| **5.** | N6 regeneration medium (With TDZ) | N6 salts and vitamins, 0.3 g/l casein hydrolysate, 30 g/l sucrose, 1.5 mg/l NAA, 3 mg/l BAP, 4 g/l, 1 mg/l TDZ, phytagel, pH 5.8 |
| **6** | N6 regeneration medium (Without TDZ) | N6 salts and vitamins, 0.3 g/l casein hydrolysate, 30 g/l sucrose, 1.5 mg/l NAA, 3 mg/l BAP, 4 g/l phytagel, pH 5.8 |
| **7.** | ½ MS medium | MS salts and vitamins (1/2 concentration), 0.15 g/l casein hydrolysate, 20 g/l maltose, 4 g/l phytagel, pH 5.8. |

**Table S13 | List of selected genes and primers used for RT-PCR analysis of different developmental stages in *japonica* and *indica* subspecies.**

| Locus ID | Primers |
|---|---|
| **OS12G0597800** | F: 5'-TCCCAACAAGAACGGAAGAG-3'<br>R: 3'-GTTAAGCCGTGTGTTTCTGC-5' |
| **OS11G0223400** | F:5'-ACTATGGCGGAAACTTGGTG-3'<br>R: 3'-TGCTCCTCAAAATCCTCCTC-5' |
| **OS08G0461400** | F: 5'-TTCAGGCTCCAAATCTCCAG-3'<br>R: 3'-TTGCTTCCTCAAGGAACGTG-5' |
| **OS01G0947700** | F:5'-TACGTGCACAATCAGGAAGG-3'<br>R: 3'-ATGGCATCGAAGAGGTTCTG-5' |
| **OS12G0476200** | F: 5'-TCATCAAGCGAGTGATCCAG-3'<br>R: 3'-TTGATGAGAAGGCCGTAGAG-5' |
| **OS01G0197700** | F:5'-TCCTCCTCATCCTTCTTCTC-3'<br>R: 3'-TCCATCCTCTTGTCAACGTC-5' |
| **OS10G0187701** | F:5'-AGCAAGCAAAGAGTGCCAAG-3'<br>R: 3'-GGCAGTCGTCCAAGAAAAAC-5' |
| **OS09G0403967** | F: 5'-TGCCACGGGAGGGCTCGACG-3'<br>R: 3'-TTCCCTCCCGCTGGATCCGG-5' |
| **OS11G0257700** | F:5'-ATTCGGTGCCAATACACTCC-3'<br>R: 3'-GGAAGAAATGGACGCTGTTG-5' |
| **OS06G0575400** | F: 5'-AACGTCTGATGGGGATGAAC-3'<br>R: 3'-TGCAAGCTCCATCATCACTC-5' |
| **OS12G0217400** | F:5'-CAATGGAGAACGATGGTGTG-3'<br>R: 3'-CGATCATAGTGAGTGCTTGC-5' |
| **OS01G0674150** | F: 5'-CGATCGCCCTCTACTTTCTG-3'<br>R: 3'-CTTAGCTTCACCCGATCGAC-5' |
| **OS08G0195400** | F:5'-GGTGCTTCTGCTTCACCTTC-3'<br>R: 3'-AAGCTTCACCGGACTTAACC-5' |
| **OS11G0532200** | F: 5'-AGGGTTCTTGCAGAAGGTTG-3'<br>R: 3'-AGCAAAAGCTGTTCCTCCAC-5' |
| **OS03G0333400** | F:5'-TCGACGATCAACATGGACAG-3'<br>R: 3'-ACTTCTCCCTGCGGTACTTG-5' |
| **OS02G0108700** | F:5'-TGGAGCTGAGTCAAATGGTG-3'<br>R: 3'-TTCATTCTCCTGGCATCTCC-5' |
| **OS08G0442200** | F:5'- TACTTCACCGACAGCAGCAC-3'<br>R: 3'- TTCGCGTCGTACTTCATCAG-5' |
| **OS02G0141100** | F:5'- TGACATCCTGCTTCTTGGTG-3'<br>R: 3'- TCCAAGCTCATCTGTTGCAC -5' |
| **OS01G0177400** | F:5'- TCTTCTCCAAGCTCATGTGG-3'<br>R: 3'- TGTGAAACTCCTCCATCACG -5' |
| **OS03G0856700** | F:5'- GCTACTGCCACGAGATGAGC-3'<br>R: 3'- AAGAATCGCCGGAAGTAGTG -5' |
| **OS02G0830200** | F:5'- AGATTCCGGTGGTGCTAATG-3'<br>R: 3'- TTCATGATGACGCGGTTG -5' |
| **OS04G0504500** | F:5'- AGAGGTGCATCCATTTACCG-3'<br>R: 3'- TTGTTCCCTGCAACTCTTCC -5' |
| **Actin (Rice)** | F:5'- GAGTATGATGAGTCGGGTCCAG-3'<br>R: 3'- ACACCAACAATCCCAAACAGAG -5' |
